# STAT3 Enhances Sensitivity of Glioblastoma to Drug-Induced Autophagy-Dependent Cell Death

**DOI:** 10.3390/cancers14020339

**Published:** 2022-01-11

**Authors:** Janina Remy, Benedikt Linder, Ulrike Weirauch, Bryan W. Day, Brett W. Stringer, Christel Herold-Mende, Achim Aigner, Knut Krohn, Donat Kögel

**Affiliations:** 1 Neuroscience Center, Experimental Neurosurgery, Department of Neurosurgery, Goethe University Hospital, 60590 Frankfurt am Main, Germany; Janina.Remy@gmx.de (J.R.); linder@med.uni-frankfurt.de (B.L.); 2 Rudolf-Boehm-Institute for Pharmacology and Toxicology, Clinical Pharmacology, University of Leipzig, 04103 Leipzig, Germany; Ulrike.Weirauch@medizin.uni-leipzig.de (U.W.); Achim.Aigner@medizin.uni-leipzig.de (A.A.); 3 Sid Faithful Brain Cancer Laboratory, QIMR Berghofer, Herston, QLD 4006, Australia; bryan.day@qimrberghofer.edu.au; 4 College of Medicine and Public Health, Flinders University, Sturt Rd., Bedford Park, SA 5042, Australia; brett.stringer@flinders.edu.au; 5 Division of Experimental Neurosurgery, Department of Neurosurgery, University Hospital Heidelberg, INF400, 69120 Heidelberg, Germany; Christel.Herold-Mende@med.uni-heidelberg.de; 6 Core Unit DNA-Technologies, IZKF, Faculty of Medicine, University of Leipzig, 04103 Leipzig, Germany; Knut.Krohn@medizin.uni-leipzig.de; 7 German Cancer Consortium DKTK Partner Site Frankfurt/Main, 60590 Frankfurt am Main, Germany; 8 German Cancer Research Center DKFZ, 69120 Heidelberg, Germany

**Keywords:** STAT3, glioblastoma, pimozide, autophagy, autophagy-dependent cell death, lysosome, lysosomal-dependent cell death

## Abstract

**Simple Summary:**

Glioblastoma is the most common primary brain cancer in adults. One reason for the development and malignancy of this tumor is the misregulation of certain cellular proteins. The oncoprotein STAT3 that is frequently overactive in glioblastoma cells is associated with more aggressive disease and decreased patient survival. Autophagy is a form of cellular self digestion that normally maintains cell integrity and provides nutrients and basic building blocks required for growth. While glioblastoma is known to be particularly resistant to conventional therapies, recent research has suggested that these tumors are more sensitive to excessive overactivation of autophagy, leading to autophagy-dependent tumor cell death. Here, we show a hitherto unknown role of STAT3 in sensitizing glioblastoma cells to excessive autophagy induced with the repurposed drug pimozide. These findings provide the basis for future research aimed at determining whether STAT3 can serve as a predictor for autophagy-proficient tumors and further support the notion of overactivating autophagy for cancer therapy.

**Abstract:**

Glioblastoma (GBM) is a devastating disease and the most common primary brain malignancy of adults with a median survival barely exceeding one year. Recent findings suggest that the antipsychotic drug pimozide triggers an autophagy-dependent, lysosomal type of cell death in GBM cells with possible implications for GBM therapy. One oncoprotein that is often overactivated in these tumors and associated with a particularly dismal prognosis is Signal Transducer and Activator of Transcription 3 (STAT3). Here, we used isogenic human and murine GBM knockout cell lines, advanced fluorescence microscopy, transcriptomic analysis and FACS-based assessment of cell viability to show that STAT3 has an underappreciated, context-dependent role in drug-induced cell death. Specifically, we demonstrate that depletion of STAT3 significantly enhances cell survival after treatment with Pimozide, suggesting that STAT3 confers a particular vulnerability to GBM. Furthermore, we show that active STAT3 has no major influence on the early steps of the autophagy pathway, but exacerbates drug-induced lysosomal membrane permeabilization (LMP) and release of cathepsins into the cytosol. Collectively, our findings support the concept of exploiting the pro-death functions of autophagy and LMP for GBM therapy and to further determine whether STAT3 can be employed as a treatment predictor for highly apoptosis-resistant, but autophagy-proficient cancers.

## 1. Introduction

Glioblastoma (GBM; grade IV glioma, WHO) is a primary brain tumor that mainly occurs in adults. GBM-patients have a very dismal prognosis with a median survival of ~15 months despite aggressive radiochemotherapy after maximally possible surgery [1]. Since GBMs are characterized by their diffuse infiltrative growth, a complete surgical resection is nearly impossible and the tumors quickly recur, often with an even more aggressive growth [2]. Different molecular subtypes of GBM have been described based on the genetic profiles of the tumors. Although their nomenclature is not uniformly used, the general consensus agrees on three GBM subtypes: proneural, classical and mesenchymal [3,4], of which the latter one is the most aggressive. Signal Transducer and Activator of Transcription (STAT3) is a molecular hub-like oncoprotein that is highly overactivated in GBM and associated with the mesenchymal subtype [5,6]. STAT3 is known to regulate multiple hallmarks of cancer such as proliferation, cell survival, angiogenesis and immune evasion (reviewed in [7,8]). The activation of STAT3 is mediated by multiple upstream kinases [9] which phosphorylate the protein at two different sites (reviewed in [10], Tyrosine705 (pY705Stat3) and Serine727 (pS727Stat3). Upon activation, STAT3 forms homodimers or heterodimers with other proteins of the STAT-family, translocates into the nucleus and induces pronounced changes in gene expression. Known targets include genes responsible for various hallmarks of cancer like migration/invasion (MMP2, MMP9; [11,12]) or EMT-like features and immune evasion/suppression (e.g., SNAI1; [13,14]). Consequently, we and others previously demonstrated that targeting STAT3 by upstream signaling inhibitors of the STAT3 pathway or by genetic depletion decreases glioma cell proliferation and migration in vitro and prolongs overall survival of tumor-bearing mice in vivo [15,16,17,18,19,20,21]. Nonetheless, these approaches have so far not resulted in clinical applications, which is in part due to the lack of specific inhibitors of STAT3, which additionally need to be able to cross the blood–brain barrier.

An underappreciated function of STAT3 is its involvement in autophagy and lysosomal function. Recent studies could already show that STAT3 can transcriptionally regulate several genes relevant for autophagy [22,23,24,25,26,27]. In addition, unphosphorylated, cytoplasmic STAT3 can have direct, transcription-independent effects on the autophagy pathway [28]. Furthermore, upon translocation to the mitochondria STAT3 was found to decrease mitochondrial reactive oxygen species (ROS) that in turn may trigger autophagy [22,29,30,31,32]. Notably, STAT3 has also been reported to be important for regulating lysosomal membrane permeabilization (LMP) and lysosome-dependent cell death (LDCD) during post-lactational regression of the mammary gland epithelium [33,34] and in breast cancer cells [35]. Curiously, under these circumstances STAT3 rather promotes cell death, a function that is in stark contrast to its canonical anti-apoptotic pro-survival role.

The lysosomes have been fittingly described as the “suicide bags” of the cells, because of their low pH and content of acidic hydrolases which are able to degrade macromolecules [36]. Damage to the lysosomal membrane, for example through cationic amphiphilic drugs [37,38], can trigger lysosomal membrane permeabilization (LMP), causing lysosomal rupture and release of its content into the cytosol [39]. The concomitant release of lysosomal cathepsin proteases and hydrolytic enzymes can lead to a specific type of cell death termed lysosome-dependent cell death (LDCD), that depending on the extent of LMP can have necrotic, apoptotic or apoptosis-like features [39]. LDCD has been observed in several pathophysiological conditions such as inflammation, aging, neurodegeneration and cardiovascular disorders [37,39,40,41]. It has been reported that cancer cells hijack lysosomes to remodel the extracellular matrix in order to increase tissue invasion and metastasis, while at the same time destabilizing them [42]. Accordingly, an increased expression, activity and secretion of cathepsins, as well as changes in lysosomal volume, composition and cellular distribution have been observed in different tumors [43]. Based on the findings suggesting both an increased lysosomal activity and concomitant lysosomal destabilization in certain tumors, these organelles might represent a double-edged sword and render cancer cells more prone to lysosome-targeting agents and LDCD via excessive activation of LMP [42]. In our recent work, we could demonstrate that the repurposed drug Pimozide (Pimo) that acts as a functional inhibitor of the lysosomal enzyme acid sphingomyelinase (FIASMA) stimulates autophagy, impairs lipid trafficking/metabolism and destabilizes lysosomes to induce LMP and a unique cell death modality with characteristics of both autophagy-dependent cell death (ADCD) and lysosome-dependent cell death (LDCD) [38,44]. Based on these features, we propose the term autophagy-dependent lysosomal cell death (ADLCD) for this particular mixed type of cellular demise.

Here, we used multiple isogenic Crispr/Cas9-derived knockout and stable shRNA-mediated knockdown cell lines to show that depletion of STAT3 has strong rescuing effects on lysosomal stability and this type of cell death, suggesting that STAT3-proficient tumors are particularly vulnerable to this novel therapeutic approach.

## 2. Results

### 2.1. Knockout of STAT3 Attenuates Autophagy-Dependent Cell Death

To analyze the possible role of STAT3 in ADLCD we first tested a panel consisting of 12 human and murine GBM cell lines for their STAT3 expression level and activation state (Supplemental Figure S1A). We observed that STAT3/Stat3 expression varies between the different cell models (Supplemental Figure S1B), but the highest activation (Tyr705-phosphorylation; Supplemental Figure S1C) was found in the human GBM cell MZ-54 and the murine GBM cell line Tu9648. MZ-54 have also been previously shown to have a high expression of STAT3 [45].

**Figure 1:**
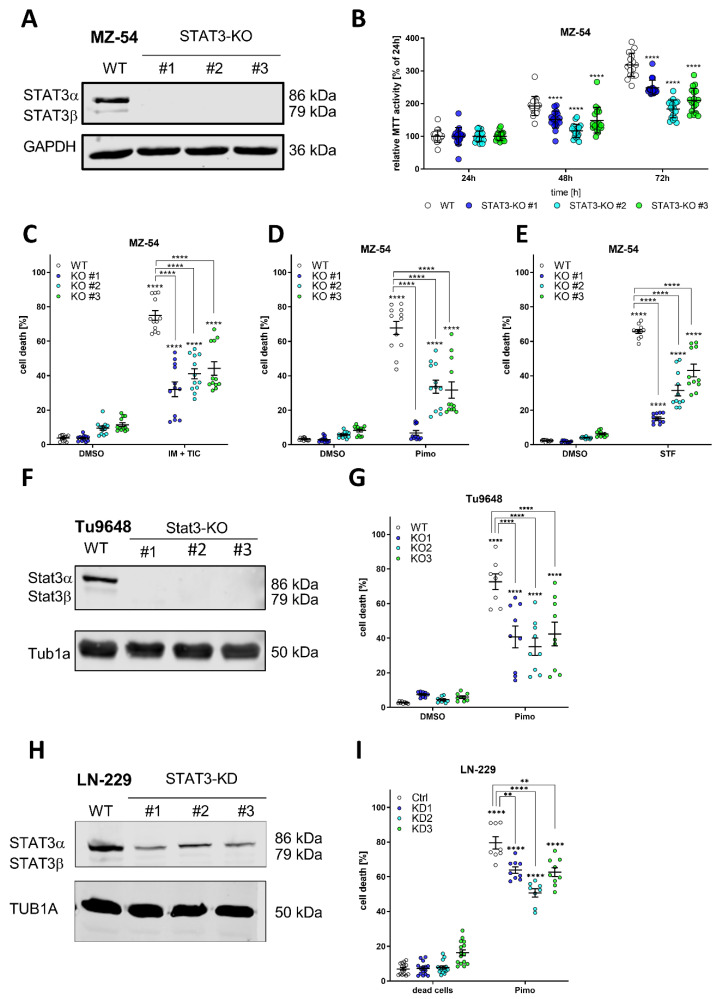
Depletion of STAT3 protects GBM cells from autophagy-dependent cell death. (**A**) Western Blot of STAT3 after Crispr/Cas9-mediated knockout (KO) of STAT3 in MZ-54 human GBM cells showing complete absence of detectable STAT3 in the KO cell lines. GAPDH served as a housekeeping protein. (**B**) MTT cell proliferation assay showing reduced growth rate of STAT3-KO cell lines compared to WT. (**C**–**E**) ADCD inducers triggered non-apoptotic cell death that could be reversed by complete loss of STAT3. MZ-54 WT and STAT3-KO cells were treated with (**C**) 20 μM imipramine (IM) and 100 μM ticlopidine (TIC), (**D**) 12.5 μM pimozide (Pimo) and (**E**) 40 μM STF-62247 (STF) for 40 h. DMSO was used as vehicle control treatment. Total cell death was determined by subsequent Annexin-V-APC/PI staining and FACS analysis. (**F**) Western Blot of Cripsr/Cas9-mediated KO of Stat3 of the murine GBM cell line Tu9648. (**G**) Tu9648 WT and Stat3-KO cells were treated with 7.5 µM Pimo for 40 h and cell death was determined. (**H**) Western Blot of LN-299 human GBM cells after stable depletion of STAT3 using shRNA. (**I**) LN-229 Ctrl and STAT3-KD cells were treated with 12.5 µM Pimo for 40 h and cell death was determined. The data are presented as point plots of at least three experiments performed with 4 replicates. ** *p* < 0.01; **** *p* > 0.0001; Two-Way ANOVA with Tukey’s multiple comparison test (GraphPad Prism 9).

Hereafter, we generated STAT3-knockouts via Crispr/Cas9. Successful STAT3-knockouts of MZ-54 were validated via western Blot (Figure 1A). We could confirm the expected limiting effects of the STAT3-KO on several hallmarks of cancer, including proliferation (analyzed by MTT assays; Figure 1B), cell migration (analyzed by scratch migration assays; Supplemental Figure S2A,B) and cell invasion (analyzed by sphere invasion assays into matrigel; Supplemental Figure S2C,D). Lastly, we confirmed decreased gene expression of the known STAT3 target genes [17,18,24,25,26,46,47] Hypoxia Inducible Factor 1 Alpha (HIF1A), Matrix Metalloprotease 1 (MMP1), MMP2, MMP9, Snail Family Transcriptional Repressor 1 (SNAI1, previously known as Snail), BCL2/adenovirus E1B 19 kDa protein-interacting protein 3 (BNIP3) and BNIP3-like (BNIP3L) as well as Solute Carrier Family 2 Member 1 (SLC2A1, also referred to as GLUT1) and SLC2A3 (also referred to as GLUT3), after depletion of STAT3 (Supplemental Figure S2E).

After successfully confirming the biological applicability of these STAT3-KO cells we analyzed whether loss of STAT3 may influence the sensitivity of the cells to pro-death autophagy and LMP by using recently described ADLCD-inducing drugs consisting of (1) a combination treatment of imipramine and ticlopidine (IM + TIC) [48], which served as a positive control throughout the experiments; (2) Pimozide (Pimo) and (3) STF-62247 (STF) [38,49]. Accordingly, either treatment led to reduced cell death induction in all three KO-lines, indicating that loss of STAT3 mitigates this cell death response. Similar findings could be obtained using stable, lentiviral depletion of STAT3 using shRNA in MZ-54 human GBM cells (Supplemental Figure S3). Using the murine Tu9648 GBM cell line and its Crispr/Cas9-mediated Stat3-Kos (Figure 1F) we could confirm that Pimo-induced cell death (Figure 1G) was highly reduced compared to Stat3-proficient cells. Lastly, we tested another human GBM cell line, LN-229, which displays lower STAT3-activation compared to MZ-54 and Tu9648 and generated stable lentiviral knockdowns of STAT3 (Figure 1H). Again, we could show that Pimo-induced cell death is enhanced by STAT3 expression (Figure 1I).

In addition, we re-expressed STAT3 using stable lentiviral overexpression in MZ-54 STAT3-KO cells (Supplemental Figure S4A,B) and could show that this leads to a re-sensitization of Pimo-induced cell death (Supplemental Figure S4C). Lastly, considering that STAT3-activity has been previously associated with the mesenchymal subtype of GBM [5,6] we analyzed if spheroid cultures, so called glioblastoma stem-like cells (GSC), representing the different molecular subtypes show differential sensitivity to Pimo (Supplemental Figure S5). For this purpose, we performed MTT assays of 4 mesenchymal GSCs (FPW1, MN1, RKI1, RN1 [50]), 3 classical GSCs (NCH1425, NCH601 [51], PB1 [50]) and 3 proneural GSCs (NCH644, NCH421k [52], MMK1 [50]) after treatment with 10, 12.5 or 15 µM Pimo for 24 or 48 h, respectively. We observed that after 24 h (Supplemental Figure S5A) the mesenchymal and classical GSC lines show an enhanced sensitivity towards Pimo, since higher concentrations (>12.5 µM) were needed to reach statistical significance of MTT activity changes. This is further corroborated after 48 h (Supplemental Figure S5B) with an effective inhibition in both mesenchymal and classical GSCs using either concentration, whereas proneural GSCs still retain a considerable amount of metabolic activity.

**Figure 2:**
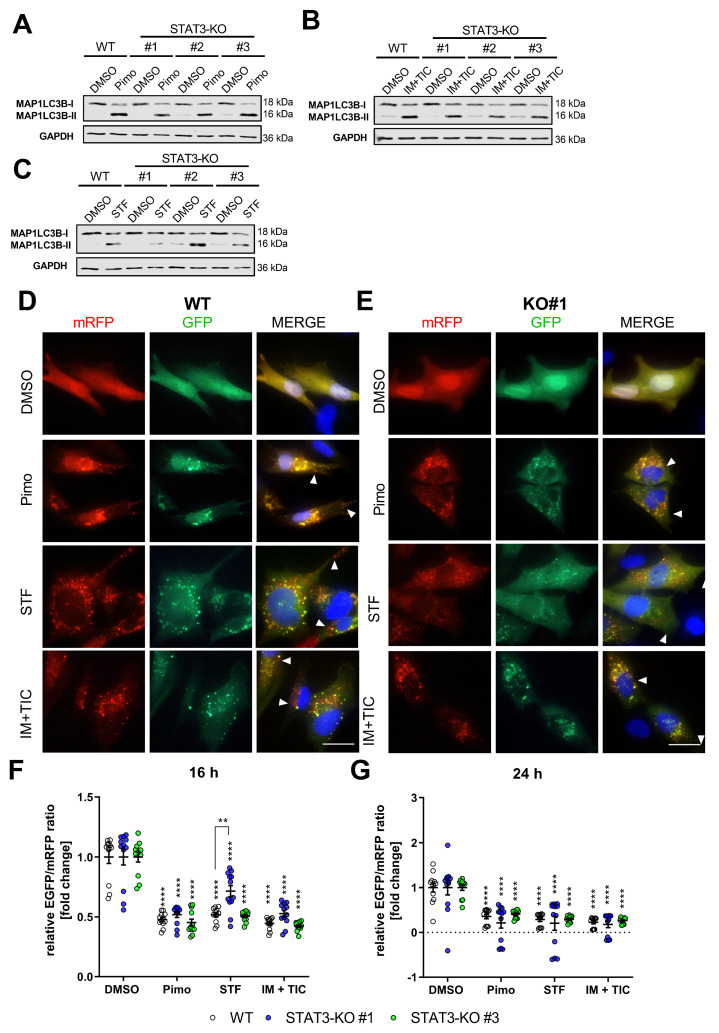
Depletion of STAT3 does not affect autophagy induction or autophagic flux. (**A**–**C**) Representative Western Blots of MAP1LC3B-I and II of MZ-54 WT cells and STAT3-KO-cells after treatment with (**A**) 12.5 µM Pimozide (Pimo), (**B**) 20 µM imipramine (IM) and 100 µM ticlopidine (TIC) and (**C**) 40 µM STF-62247 (STF) for 6 h. GAPDH served as a housekeeping protein. (**D**,**E**) Representative pictures of (**D**) MZ-54 WT cells and (**E**) STAT3-KO#1 cells stably transfected with ptf-LC3 for the expression of mRFP-GFP-MAP1LC3B and treated with 15 µM Pimo, 40 µM STF, 20 µM IM and 100 µM TIC or vehicle (DMSO) for 24 h. Note that the presence of red-only punctae indicating active flux is unchanged between WT and STAT3-KO cells (arrow heads). Scale bar: 25 µm. (**F**,**G**) Flow cytometric quantification of the autophagic flux using stably transfection of pMRX-IP-GFP-LC3-RFP-LC3ΔG of MZ-54 WT and STAT3-KO cells. ** *p* < 0.01; **** *p* > 0.0001; Two-Way ANOVA with Tukey’s multiple comparison test (GraphPad Prism 7).

Since changes in ADCD-induction could be caused by a perturbation of the autophagic flux, we next assessed if STAT3-depletion may directly affect it. For this purpose we analyzed the conversion of MAP1LC3B-I (LC3-I) to MAP1LC3B-II (LC3-II) after treatment with Pimo (Figure 2A), STF (Figure 2B,D) and IM + TIC (Figure 2C) and found that in STAT3-WT and STAT3-KO cells all ADLCD-inducing drugs caused a pronounced shift from LC3-I to LC3-II that was independent from the presence of STAT3. We therefore concluded that STAT3 has no major effect on autophagy induction.

To further analyze the functionality of the autophagy pathway, we analyzed the autophagic flux using the LC3-mRFP-GFP tandem construct (ptf-LC3; Addgene #21074; [53]). Therefore, stable transfectants were generated and analyzed via immunofluorescence after treatment. The autophagic flux can be analyzed because this double-tagged LC3 can be attached to autophagosomes, indicative of autophagy induction, while the EGFP-signal will be quenched after fusion of autophagosomes with lysosomes resulting in mRFP-only positive autolysosomes. By analyzing the ratio of green to red dots, the induction and proceeding of autophagy (i.e., the flux) can be quantified. Accordingly, we observed a pronounced induction of autophagy in both WT and STAT3-KO#1 (Figure 2D) as well as STAT3-KO#2 and #3 (Supplemental Figure S6A,B) cells as shown by multiple double-positive punctae per cell after treatment with either compound. Additionally, treatment with all drugs was further associated with the presence of red-only punctae, indicating successful fusion of autophagosomes with lysosomes. This was further confirmed after quantifying and calculating the ratio of green and red fluorescence intensity of cells stably transfected with the plasmid pMRX-IP-GFP-LC3-RFP-LC3ΔG (Addgene #84572; [54]) and measurement using a flow cytometer (Figure 2F,G). These experiments showed a decreased EGFP/mRFP-ratio, indicative of an active autophagic flux. Collectively, these experiments led us to conclude that STAT3 has no major influence on the autophagic flux in GBM cells.

### 2.2. Knockout of STAT3 Confers Sensitivity to Lysosomal Membrane Permeabilization

**Figure 3:**
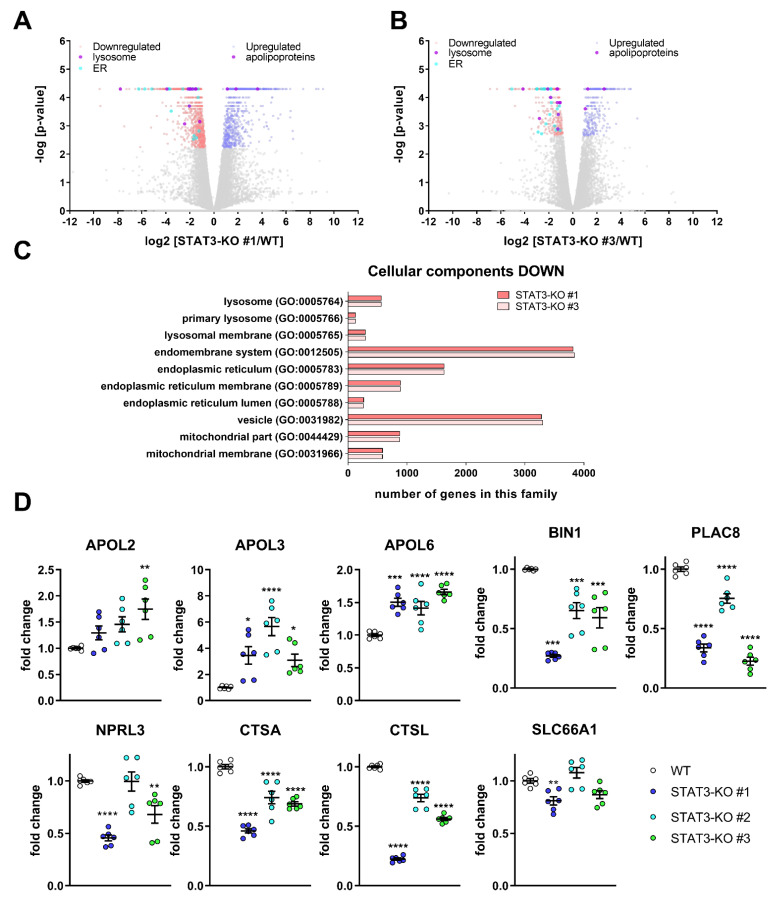
Transcriptomic analyses after STAT3 depletion in MZ-54-cells reveal changes in lysosomal genes and lipid metabolism and validation of selected target genes. (**A**,**B**) Volcano Plots of (**A**) STAT3-KO#1 vs. WT and (**B**) STAT3-KO#3 vs. WT displaying significantly decreased (red) and increased (blue) transcripts. Transcripts related to the lysosome (violet, left side) and ER (cyan, left side) as well as apolipoproteins (violet, right side) are displayed among the significantly regulated transcripts. (**C**) Bar graphs of selected GO Cellular Component processes after performing an enrichment analysis using PANTHER (Version 14.1, [56]). (**D**) Point Plots of Taqman-based gene expression analyses of the selected high-ranking candidates APOL2, APOL3, APOL6, BIN1, PLAC8, NPRL3, CTSA, CTSL and PQLC2 from the whole transcriptome analyses of MZ-54 WT cells and STAT3-KO cells. Summary of two experiments performed in triplicates. * *p* < 0.05; ** *p* < 0.01; *** *p* < 0.001; **** *p* > 0.0001; One-Way ANOVA with Dunnetts multiple comparison test (GraphPad Prism 7).

In order to systematically investigate the potential underlying effects of STAT3-depletion on drug sensitivity, we took two of the STAT3-KO lines and compared their transcriptomic profiles with control cells under basal, i.e., untreated conditions. We found that in both KO lines, 141 transcripts were significantly decreased and 140 increased compared to WT (Figure 3A,B). Except for STAT3, which was significantly depleted, no other STAT family members were significantly changed. An enrichment analysis (Figure 3C) showed that many lysosomal processes such as lysosome (GO:0005764), primary lysosome (GO:0005766) and lysosomal membrane (GO:0005765) were reduced. Additionally, many processes related to the ER were significantly reduced. The transcriptome analysis further revealed an upregulation of cholesterol transport-mediating Apolipoproteins L (APOLs) (Figure 3D) and a decrease in lipid metabolic processes (Supplemental Figure S7A,B), that have been implicated in cholesterol transport [55] in the absence of STAT3. Recently, we showed that the proteomic profiles of MZ-54 cells treated with the ADLCD-inducing drugs Pimo and IM + TIC displayed very similar changes [38], in particular in lipid metabolic processes (most pronounced in cholesterol metabolism) and lysosomal function. In line with the proteomic data, cholesterol accumulation in the lysosomes and perturbed lysosomal function including lysosomal membrane permeabilization (LMP) and ADLCD were observed after treatment with these drugs [38].

Given that the drugs used in this study have been previously shown to affect lipid metabolic processes and lysosomal function [38,57] and that these processes are also altered in STAT3-KO cells, we validated selected high-ranking candidate genes by qPCR using MZ-54 with STAT3-KO (Figure 3D) and additionally with STAT3-KD (Supplemental Figure S7C). Of those, the three APOL-transcripts APOL2, APOL3 and APOL6, which have been implicated in cholesterol transport [55], were successfully validated. Among the decreased transcripts, we validated (1) BIN1, which is a potential tumor suppressor [58] and is related to the GO-term “endosome to lysosome transport” (GO:000833), (2) PLAC8, that facilitates fusion of autophagosome and lysosomes [59] and (3) NPRL3, which is a lysosomal protein and part of the process “regulation of autophagosome assembly” (GO:2000785). Lastly, we confirmed that the transcripts of the cathepsins A and L (CTSA and CTSL) are decreased in STAT3-depleted cells, whereas the amino acid transporter SLC66A1 can only be considered as moderately reduced in two of the three STAT3-KO clones. Using the GlioVis-portal [60] we correlated STAT3 expression with the expression of these genes derived from TCGA-data [61] across the three different subtypes and without subtyping (Supplementary Figure S8; Supplementary Table S1). We observed that the three APOL-transcripts APOL2, APOL3 and APOL6 (Supplementary Figure S8A–C) are positively correlated with STAT3 across all GBM samples and, except APOL3, also in mesenchymal GBM samples. BIN1, PLAC8 and NPRL3 (Supplementary Figure S8D–F) were not correlated with STAT3 among all GBM, whereas a positive, negative and positive correlation was observed for mesenchymal samples, respectively. CTSA was also positively correlated with STAT3, irrespective of subtype (Supplementary Figure S8G), while CTSL was not included in the dataset. Lastly, SLC66A1 (Supplementary Figure S8H) did not show any correlation with STAT3.

**Figure 4:**
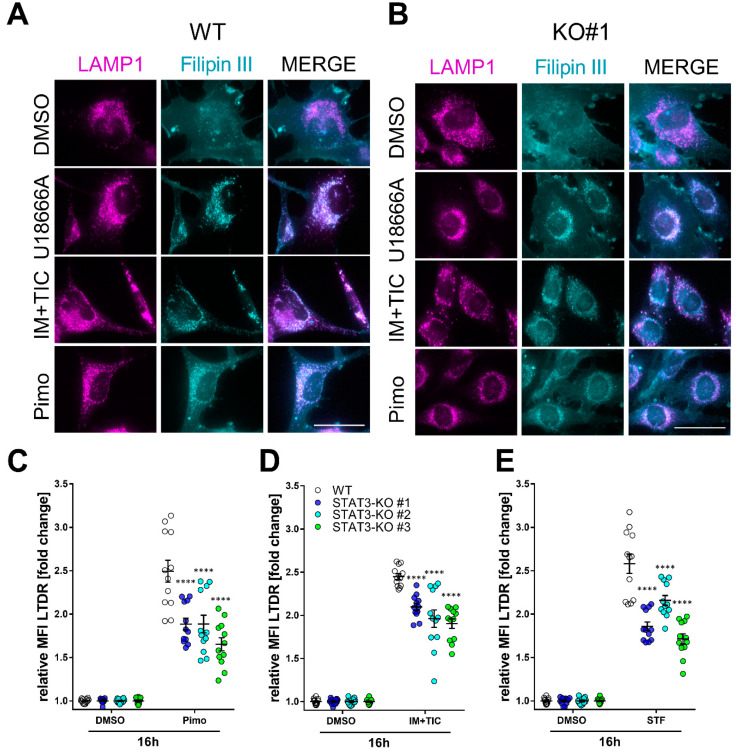
Depletion of STAT3 reduces lysosomal cholesterol accumulation and lysosomal acidification. (**A**,**B**) Representative pictures of (**A**) MZ-54 WT cells and (**B**) STAT3-KO#1 cells stained with Filipin III and against LAMP1 after treatment with 15 µM Pimozide (Pimo), 40 µM STF-62247 (STF), 20 µM imipramine (IM) and 100 µM ticlopodine (TIC) or vehicle (DMSO) for 18 h. Scale bar: 50 µm. As a positive control the cells were treated with 1.25 µM of the cationic, amphiphile U18666A that inhibits intracellular cholesterol trafficking [62]. Note that white punctae in the merged image indicates cholesterol accumulation within the lysosomes, which is reduced in STAT-KO cells. (**C**–**E**) Quantification of lysosomal acidification of MZ-54 WT and STAT3-KO cells using lysotracker deep red after treatment as in (**A**,**B**) for 16 h. The data are presented as point-plots of three experiments performed in 4 replicates. **** *p* < 0.0001; Two-way ANOVA with Sidak’s multiple comparison (GraphPad Prism 7).

To investigate how lipid trafficking and lysosomal accumulation may be affected by STAT3-depletion we decided to analyze lysosomal cholesterol accumulation as a surrogate for lipid accumulation. For this purpose, we analyzed the cells 18 h after treatment with IM + TIC, Pimo, STF, solvent (DMSO) or the positive control U18666A (Figure 4), a cationic, amphiphilic drug that inhibits intracellular cholesterol trafficking [62] and staining with the cholesterol-dye Filipin III in combination with the lysosomal marker LAMP1. In MZ-54 WT cells (Figure 4A) we observed a profound colocalization of Filipin III and LAMP1 upon treatment with IM + TIC and Pimo. The three STAT3-KO clones (Figure 4B and Supplemental Figure S9A,B) exhibited decreased colocalization upon treatment with IM + TIC and Pimo, indicating an alleviating effect of the STAT3 KO on lysosomal cholesterol accumulation. Accordingly, the flow cytometric quantification of Lysotracker Deep Red (LTDR; Figure 4C–E) shows that STAT3 depletion also significantly reduced the amount of acidic vacuoles in all three cell lines.

**Figure 5:**
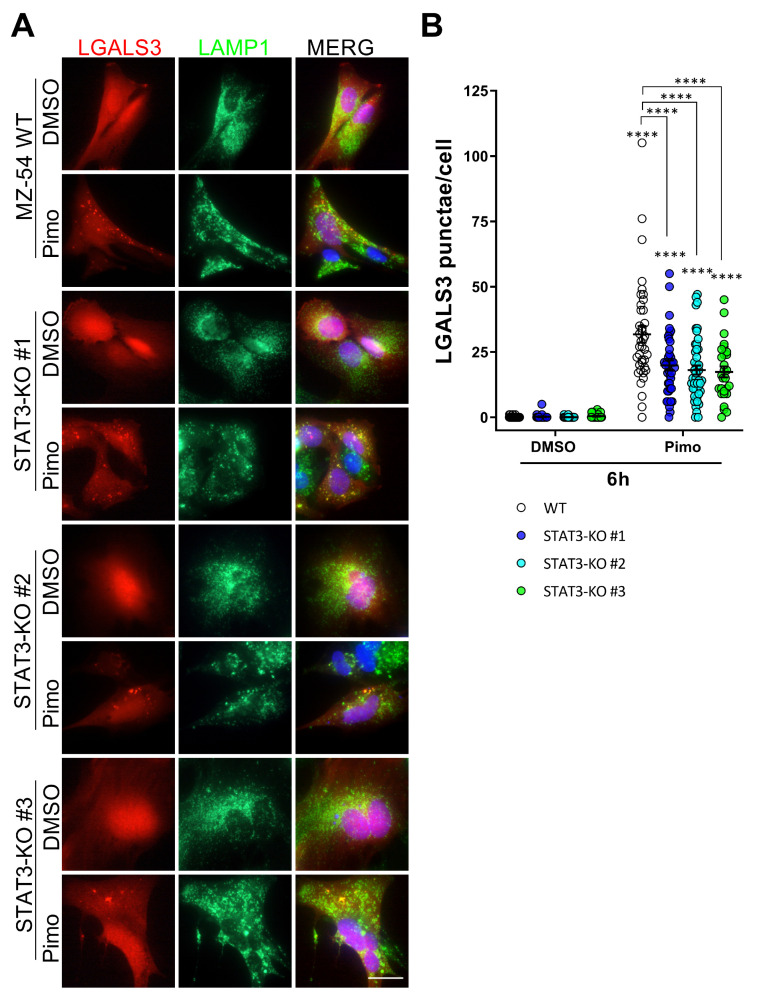
Depletion of STAT3 reduces lysosomal membrane permeabilization and release of lysosomal content. (**A**) Representative pictures of stably transfected MZ-54 WT and STAT3-KO cells expressing LGALS3-mCherry and co-stained against the lysosomal marker LMAP1 after treatment with 25 µM Pimozide (Pimo) or vehicle (DMSO) for 6 h. Note that MZ-54 WT cells exhibit more yellow punctae in the merged view indicating LMP. (**B**) Quantification of LGALS3-punctae of cells treated as in (**A**) depicted as point plots of three experiment of which at least 3 vision fields were counted (in total 29 to 48 cells per condition). **** *p* > 0.0001; Two-Way ANOVA with Tukey’s multiple comparison test (GraphPad Prism 7).

These changes in lysosomal function are in agreement with previous reports showing an involvement of STAT3 in LMP and LDCD [33,34,35]. To investigate LMP in more detail, we established stable Galectin3 (LGALS3)-mCherry-expressing MZ-54 cells using the pmCherry-Gal3 plasmid (Addgene #85662; [63]). LGALS3 specifically translocates to leaky membrane of lysosomes [63,64]. Upon co-staining with LAMP1 (Figure 5A) as a lysosomal marker several LGALS3-positive punctae were observed after treatment with Pimo in WT cells, which colocalize with LAMP1 [38]. In STAT3-KO cells we can also observe LGALS3-punctae, but to a lesser extent. Upon quantification (Figure 5B), this decrease was found to be statistically significant indicating a reduced rupture of lysosomal membranes in STAT3-KO cells.

**Figure 6:**
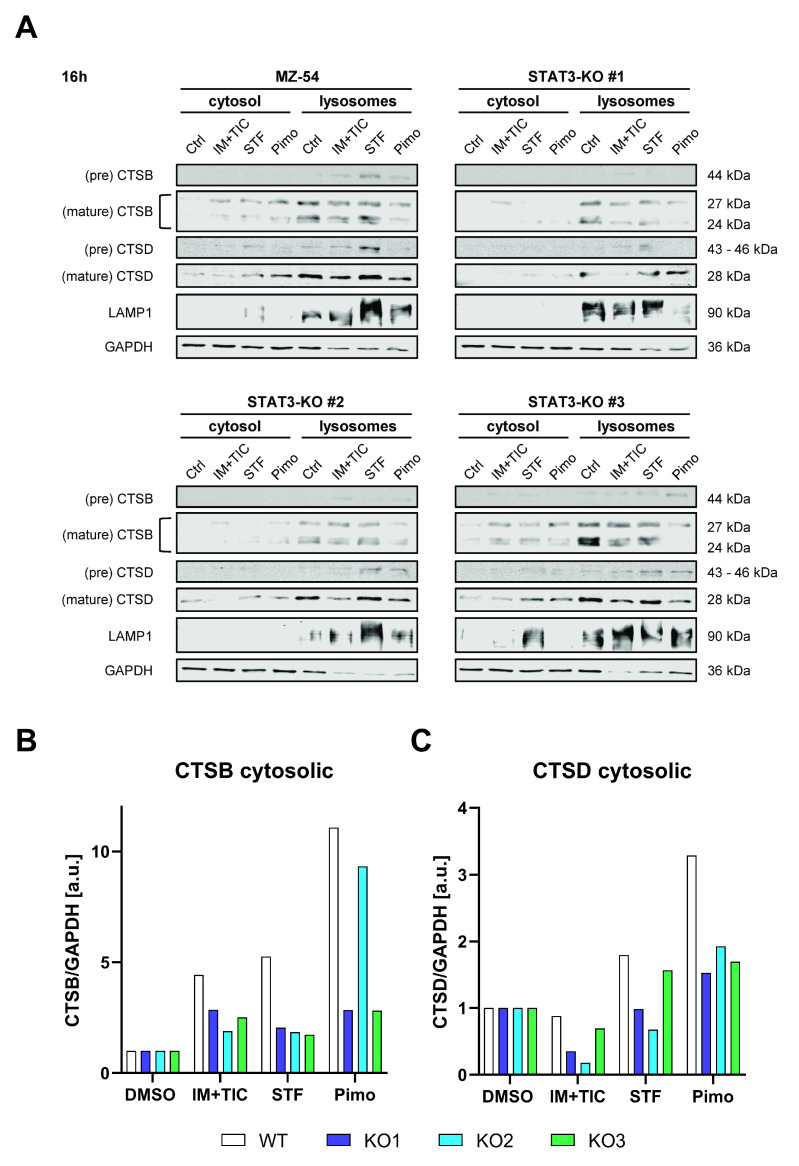
Depletion of STAT3 reduces lysosomal membrane permeabilization and release of lysosomal content. (**A**) Western Blots of MZ-54 WT and STAT3-KO cells after digitonin-based fractionation of cytosolic and lysosomal proteins 16 h after treatment with 20 µM imipramine (IM) and 100 µM ticlopodine (TIC), 15 µM Pimozide (Pimo), 40 µM STF-62247 (STF) or vehicle (DMSO). LAMP1 and GAPDH served as housekeeping proteins for the lysosomal and cytosolic fraction, respectively. (**B**,**C**) Quantification of the cytosolic fraction of (**B**) CTSB and (**C**) CSTD. The data for CTSB and CTSD were first normalized to GAPDH and then to DMSO for each cell line, respectively.

One key characteristic of LMP is the subsequent leakage of lysosomal content, such as cathepsins into the cytosol. Accordingly, we performed digitonin-mediated fractionation of the cytosolic and lysosomal compartments after treatment with IM + TIC, STF or Pimo and detected cathepsin B (CTSB) and D (CTSD) via Western Blot. In WT cells both proteins were found to be prominently enriched in the cytosolic fraction after treatment, whereas the amount of released cathepsins was considerably reduced in at least two out of three knockout lines (Figure 6).

**Figure 7:**
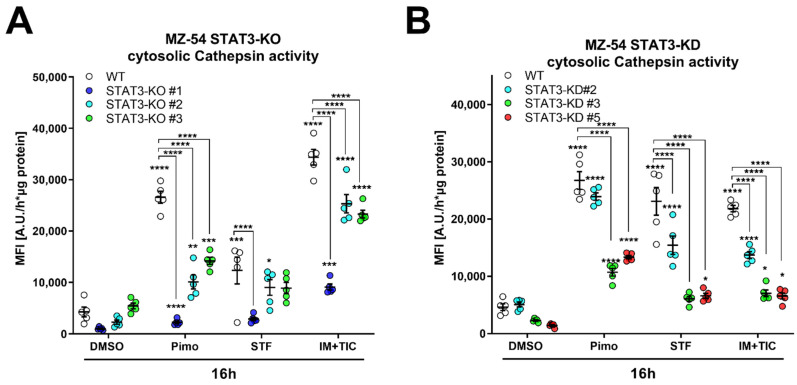

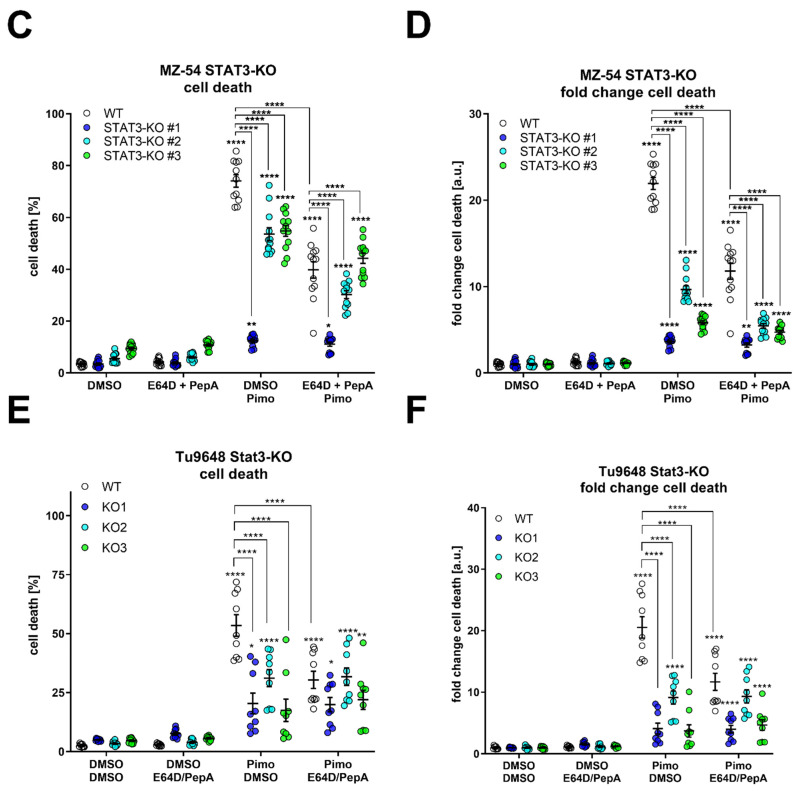
Depletion of STAT3 reduces cytosolic cathepsin activity and inhibition of cathepsins attenuates cell death. (**A**,**B**) Cytosolic CTSB activity of (**A**) MZ-54 WT and STAT3-KO cells and (**B**) MZ-54 shCtrl and STAT3-KD cells 16 h after treatment with 20 µM imipramine (IM) and 100 µM ticlopodine (TIC), 15 µM Pimozide (Pimo), 40 µM STF-62247 (STF) or vehicle (DMSO) after cytosolic fractionation (for details see materials and methods section). (**C**) Total cell death was determined by Annexin-V-APC/PI staining and FACS analysis after treatment of MZ-54 WT and STAT3-KO cells after treatment with with 12.5 µM Pimozide (Pimo) or vehicle (DMSO) with and without additional treatment with the cathepsin-inhibitors E64D (25 µM) and pepstatin A (PepA, 50 µM). (**D**) Fold change cell death from the data presented in C. The data are baseline-corrected to each DMSO-treated cell line in order to adjust for baseline cell death-induction after STAT3-KO. (**E**) Total cell death was determined by FACS analysis of Tu9648 WT and Stat3-KO cells after treatment with 7.5 µM Pimo for 40 h with and without cathepsin-inhibitors. (**F**) Baseline-correction of the data presented in (**E**). The data are presented as point plots of (**A**,**B**) 2 experiments performed in 5 replicates and (**C**–**F**) three experiments performed 3–4 replicates. * *p* < 0.05; ** *p* < 0.01; *** *p* < 0.001; **** *p* > 0.0001; Two-Way ANOVA with Tukey’s multiple comparison test (GraphPad Prism 7).

In order to obtain more quantifiable data, we measured CTSB activity in the cytosolic fraction using a fluorescence-based enzyme kinetic assay (Figure 7). For this experiment, we treated MZ-54 WT and STAT3-KO cells (Figure 7A), as well as MZ-54 shCtrl and STAT3-KD cells (Figure 7B) with Pimo, STF and IM + TIC, and performed digitonin-based fractionation. We found that in WT and shCtrl cells all three treatments evoked significant increases of cytosolic CTSB activity, indicating LMP-induced leakage of lysosomal content into the cytosol. Complete loss of STAT3 (KO) and reduction in STAT3 expression (KD) significantly reduced the extent of cytosolic CTSB activity, sometimes even close to baseline values. Lastly, we reasoned that cathepsin release, which is increased in WT cells, contributes to this cell death modality. Accordingly, pre-treatment with cathepsin inhibitors E64D and pepstatin A prevented Pimo-induced cell death (Figure 7C), in particular in WT cells (Figure 7D), and to a lesser degree in STAT3-KO cells. Although we observed a conclusive trend towards reduced cell death in STAT3-KO cells, we also noticed a basal increase in cell death of the STAT3-KO cells, especially those that seemed to be the least protected from Pimo-induced cell death. Therefore, we calculated the fold change cell death (Figure 7D) for each cell line. Using this approach, we revealed that indeed all STAT3-KO cells are similarly and strongly protected from cell death induction, while the additional cathepsin-inhibition did not confer an additional survival advantage, likely because of the reduced cathepsin release. We therefore concluded that STAT3-depletion alleviates LMP and ensuing cathepsin-dependent cell death. Similar results could be confirmed in the murine GBM cell model Tu9648, which also display profound inhibition of Pimo-induced cell death in WT cells, upon cathepsin-inhibition (Figure 7E) as well as after calculating the fold change cell death induction (Figure 7F).

## 3. Discussion

The oncogenic transcription factor STAT3 acts as a signaling hub molecule involved in regulation of most, if not all hallmarks of cancer, including proliferation, tumor invasion, altered cellular metabolism, angiogenesis, immune evasion and cell survival. Overactivation of STAT3 is frequently observed in malignant gliomas and correlated to the mesenchymal subtype of GBM, a particularly aggressive and treatment-resistant molecular subgroup of these tumors [9,65,66]. Therefore, STAT3 represents a very interesting target for GBM therapy. However, STAT3 and other oncogenic transcription factors are a unique class of targets that are very difficult to drug. Therefore, many of the current efforts are focusing on the pharmacological inhibition of upstream JAK kinases in the JAK/Stat pathway, including a phase I clinical trial with the JAK inhibitor WP1066 (NCT01904123), although it should be noted that non-receptor tyrosine kinases such as bone marrow and X-linked (BMX) and members of the Src family can bypass JAK-dependent activation of STAT3 in GBM [9,67]. An alternative approach to target STAT3-driven GBMs is to identify and exploit particular vulnerabilities of these tumors. Extensive and prolonged overactivation of autophagy and the concomitant induction of ACD represents a particular sensitivity of GBM cells and an interesting new therapeutic approach [48,57,68,69,70]. While STAT3 has been found to be important for the (positive and negative) regulation of autophagy [32], the exact role of STAT3 in autophagy is highly dependent on the subcellular localization of STAT3 and the cellular context [32].

Recently, we demonstrated that the ACD-inducing compounds Pimo and the combination of IM + TIC, all three being clinically approved drugs that act as “functional inhibitors of the lysosomal enzyme acid sphinogomyelinase” (FIASMAs), severely impair the trafficking/metabolism of cholesterol and other lipids and induce robust lipid-ROS formation to destabilize lysosomal membranes, thereby triggering lysosomal dysfunction and a type of cell death that is dependent both on autophagy and LMP in GBM cells [38]. The current study is a follow-up on this work and we now demonstrate that activated STAT3 significantly increases the sensitivity towards these ACD-inducing drugs, with STAT3-proficient GBM cells showing an increased LMP-mediated leakage of lysosomal content such as cathepsins into the cytosol in comparison to STAT3-depleted cells. This observation suggests that overactivated STAT3 may be useful as a biomarker for ACD susceptibility, a notion that should be investigated in further detail in future studies.

Mechanistically, this death-sensitizing role of STAT3 appears to be independent from alterations in the early steps of the autophagy pathway, because STAT3-deficient and proficient cell lines displayed a very similar extent of LC-3-conversion and an unchanged autophagic flux in our experiments. These findings indicate that STAT3 may rather interfere at the later steps of the autophagosomal/lysosomal pathway, in particular at the lysosomal level. Consistent with this hypothesis, an LMP-promoting role of STAT3 has been documented in several previous studies, for example during mammary gland involution [33,34] as well as in breast cancer cells treated with a derivative of the natural molecule riccardin D [35]. As already outlined above, IM, TIC and Pimo are FIASMAs that accumulate within the lysosomes and induce the detachment and inactivation of acid sphingomyelinase from the lysosomal membrane [71], thereby enhancing deregulation of lipid homeostasis. This event has in turn been correlated to lysosomal stress and LMP [72,73,74]. Consequently, we did observe partially reduced LMP and release of lysosomal content of STAT3-KO cells following drug treatment compared to WT cells. Of note, lysosomes represent a potential Achilles heel of certain tumors that may be therapeutically exploited. Given the fact that tumor cells often display increased lysosomal activity, but decreased lysosomal stability, lysosomes may represent a particularly suitable target for cancer treatment, because cancer cells may in general be more prone to drug-induced lysosomal destabilization compared to normal tissue [75]. The present study supports the notion that STAT3-driven tumors may be very good candidates for such an approach and our data support the hypothesis that STAT3 has a key role in driving this increased lysosomal activity [33,34,35,76]. This increase may on the one end enhance the metastatic potential of tumors, but at the same time also sensitize them for LMP-inducing drugs. Since STAT3 overactivation generally corresponds to a particularly therapy-resistant phenotype, these observations could open new avenues for the treatment of this subgroup of tumors.

In addition to enhanced lysosomal activation, the demand for cholesterol may be a second vulnerability of (STAT3-driven) tumors, in particular GBMs that are incapable of de novo cholesterol synthesis and rely on exogenous cholesterol [77,78,79]. Hence, the combined targeting of cholesterol trafficking and lysosomal function, as demonstrated to occur with the drugs used in this study [38], appears to be an interesting strategy for the treatment of GBM. Our transcriptome analysis of STAT3-KO versus wild type cells revealed global effects on components of the endomembrane system (including ER) and genes putatively related to cholesterol transport (APOL family members), hinting at the possibility that in addition to facilitating LMP, STAT3-mediated effects on lipid metabolism and trafficking may contribute to drug sensitization.

In conclusion, our data support the concept of using FIASMAs to simultaneously overactivate autophagy and induce LMP in order to target two potential Achilles heels of GBM, i.e., lysosomal function and lipid trafficking as a novel strategy for the treatment of GBM. In light of the sensitizing function of STAT3 in this context, STAT3-driven tumors may be especially suited for such a therapeutic approach.

## 4. Materials and Methods

### 4.1. Cells and Cell Culture

Human GBM cell lines A172 (ATCC #1620), MZ-18 [80], MZ-54 [80], MZ-256 [80], MZ-304 [80], U87-MG (ATCC #HTB-14), U251-MG (RRID:CVCL_0021), U343-MG (RRID:CVCL_S471), U373-MG ATCC (RRID:CVCL_2219), LN229 (ATCC # CRL-2611) and the murine GBM lines Tu2449 and Tu-9648 [81] as well as HEK293T cells (ATCC #CRL-3216) were cultured in Dulbecco’s modified Eagle’s medium (DMEM GlutaMAX) supplied with heat-inactivated 10% FBS and 100 U/mL Penicillin 100 µg/mL Streptomycin (all from Gibco, Darmstadt, Germany) in a humidified incubator at 37 °C and 5% CO_2_. For passaging and seeding, the cells were detached using Trypsin/EDTA-solution (Gibco). The GSC lines PB1, FPW1, MN1, RKI1, RN1, MMK1 were gifted from Bryan Day and Brett Stringer [50] and the GSC lines NCH1425, NCH601 [51], NCH644, NCH421k [52] were gifted from Christel Herold-Mende. All GSCs were cultured in either DMEM/F12 or Neurobasal-A Medium supplied with Glutamax (1×), B27 Supplement (1×), 100 U/mL Penicillin 100 µg/mL Streptomycin (all from Gibco), 20 ng/mL Epidermal Growth Factor (Peprotech, Hamburg, Germany) and 20 ng/mL Fibroblast Growth Factor (Peprotech) in suspension flasks.

To generate Crispr/Cas9-knockouts various potential small guide RNAs (sgRNA) were determined using the Web-App Benchling.com and were cloned into pSpCas9(BB)-2A-Puro (px459) vector (addgene #48139) as described [82,83]. After validation of successful insertion into the plasmids the cells were transfected with a combination of two different sgRNA-containing plasmids using Lipofectamine 3000 per the manufacturers’ instructions. Then, 48 h after transfection the cells were selected with 1 µg/mL puromycin (Santa Cruz Biotechnology, Dallas, TX, USA)-containing medium for 24 h. Hereafter, the cells were seeded as single cells into 96-well-plates in order to generate isogenic cell lines. Cell lines were thereafter tested for bi-allelic deletion via genotyping PCR and the loss of protein-expression was verified using SDS-PAGE and Western Blot.

The following sequences were used to generate STAT3-KOs: sgSTAT3_A_sense: 3′-aaacCAGTGGCTGCAGTCTGTAGAC-5′, sgSTAT3_A_antisense: 3′-CACCGTCTACAGACTGCAGCCACTG-5′; sgSTAT3_B_sense: 3′-aaacTTGGCTTCTCAAGATACCTGC-5′, sgSTAT3_B_antisense: 3′-CACCGCAGGTATCTTGAGAAGCCAA-5′; sgSTAT3_C_sense: 3′-aaacGTGGGAAGAATCACGCCTTC-5′, sgSTAT3_C_antisense: 3′-CACCGAAGGCGTGATTCTTCCCAC-5′. MZ-54 STAT3-KO#1, #2 and #3 were generated after transfection of sgSTAT3_A and B; A and C; B and C, respectively.

Genotyping PCR of STAT3 was conducted using the following Primer: STAT3_fwd: 3′-GTAACGACCTCCCCTTCGC-5′, STAT3_rev: 3′-TGTTTTGTCTCAGGTCTCACCT-5′.

The following sequences were used to generate Stat3-KOs: sgStat3_A_sense: 3′-aaacGGCACCTTGGATTGAGAGTC-5′, sgStat3_A_antisense: 3′-CACCGACTCTCAATCCAAGGTGCC-5′; sgStat3_B_sense: 3′-aaacGCTGTACAGCGACAGCTTCC-5′, sgStat3_B_antisense: 3′-CACCGGAAGCTGTCGCTGTACAGC-5′; sgStat3_C_sense: 3′-aaacCATGGAGCTGCGGCAGTTCC-5′, sgStat3_C_antisense: 3′-CACCGGAACTGCCGCAGCTCCATG-5′; sgStat3_D_sense: 3′-aaacGCTGCAGCAGCTGGACACAC-5′, sgStat3_D_antisense: 3′-CACCGTGTGTCCAGCTGCTGCAGC-5′.

Tu9648 Stat3-KO#1, #2 and #3 were generated after transfection of sgStat3_A and B; B and D; C and D, respectively. Genotyping PCR of Stat3 was conducted using the following Primer: Stat3_Fwd01: 3′-GAAGCCACGTGTGTGGTAGA-5′, Stat3_Rev02: 3′-TCCTTTTCAGAGTCACCAGGG-5′. All oligonucleotides were ordered from Eurofins Scientific (Eurofins Scientific, Luxembourg, Luxembourg).

To generate stable shRNA-mediated STAT3-KD cells (pLKO.1-puro_shSTAT3; MISSION^®^ SHCLNV-NM_003150, Sigma-Aldrich, Taufkirchen, Germany) or control cells expressing non-mammalian targeting control shRNA (pLKO.1-Puro_shCtrl; MISSION^®^ SHC002, Sigma-Aldrich) 150,000 HEK293T cells were seeded into 6-well plates. After overnight incubation, cells were transfected with 2 μg plasmid DNA (pLKO.1-puro), 1.5 μg gag/pol plasmid (psPAX2, addgene #12260) and 0.5 µg VSV-G envelope plasmid (pMD2.G, addgene #12259) in 57 µL Opti-MEM and 6 µL FuGENE HD (Promega, Fitchburg, WI, USA) transfection reagent. After medium change after 6 h the viral supernatant was collected after an additional 16 h and 40 h, pooled, filtered through a 0.45 µm filter. The viral supernatant was dilutet 1:1 with fresh medium and supplied 3 µg/mL hexadimethrine bromide (polybrene; Sigma-Aldrich). Puromycin was applied at 5 µg/mL and maintained to select for positively transduced cells. psPAX2 was a gift from Didier Trono (Addgene plasmid #12260; http://n2t.net/addgene:12260; RRID:Addgene_12260, accessed on: 10 December 2021). pMD2.G was a gift from Didier Trono (Addgene plasmid #12259; http://n2t.net/addgene:12259; RRID:Addgene_12259, accessed on: 10 December 2021)

To generate cells stably expressing of mRFP-GFP-MAP1LC3B using the plasmid ptf-LC3 (addgene #21074, [53]), pMRX-IP-GFP-LC3-RFP-LC3ΔG (addgene #84572, [54]) or mGALS3-mCherry using pmCherry-Gal3 (addgene #85662, [63]) the cells were transfected with Lipfectamine 3000 as described above and selected with 1 mg/mL G418 (geneticin disulphate solution; Carl Roth GmbH + Co. KG). ptfLC3 was a gift from Tamotsu Yoshimori (Addgene plasmid #21074; http://n2t.net/addgene:21074; RRID:Addgene_21074, accessed on: 10 December 2021). pMRX-IP-GFP-LC3-RFP-LC3ΔG was a gift from Noboru Mizushima (Addgene plasmid #84572; http://n2t.net/addgene:84572; RRID:Addgene_84572, accessed on: 10 December 2021). pmCherry-Gal3 was a gift from Hemmo Meyer (Addgene plasmid #85662; http://n2t.net/addgene:85662; RRID:Addgene_85662, accessed on: 10 December 2021).

To re-express STAT3 into MZ-54 STAT3-KO, we used the plasmid pDONR223_STAT3_WT (addgene #82235; [84]) to clone the STAT3 coding sequence into the plasmid pLenti CMV Puro DEST (w118-1) (addgene #17452; [85]) using a Gateway LR Clonase Reaction (Thermo Fisher) according to the instructions of the manufacturer. The resulting plasmid pLenti CMV Puro DEST_STAT3_WT was used to generate viral supernatant. pDONR223_STAT3_WT was a gift from Jesse Boehm & William Hahn & David Root (Addgene plasmid #82235; http://n2t.net/addgene:82235; RRID:Addgene_82235, accessed on: 10 December 2021). pLenti CMV Puro DEST (w118-1) was a gift from Eric Campeau & Paul Kaufman (Addgene plasmid #17452; http://n2t.net/addgene:17452; RRID:Addgene_17452, accessed on: 10 December 2021). All cell lines were monthly tested for mycoplasma infection using the PCR Mycoplasma Test Kit II (AppliChem) and only negatively tested cells were used for experiments.

### 4.2. Compounds, Antibodies and Taqman-Probes

The following compounds were dissolved in dimethyl-sulfoxide (DMSO; Carl Roth, Karlsruhe, Germany): imipramine hydrochloride (IM; Sigma-Aldrich), ticlopidine hydrochloride (TIC; Sigma-Aldrich), Pimozide (Pimo; Sigma-Aldrich), STF-62247 (STF, Santa Cruz), ABT-737 (ABT, Santa Cruz), Etoposide (Etopo, Enzo Life Sciences, Lörrach, Germany), Fillipin III (Abcam Biochemicals, Cambridge, UK), U18666A (Abcam Biochemicals), Digitonin (Sigma-Aldrich) E64D (Biomol, Hamburg, Germany), Pepstatin A (PepA, Applichem, Darmstadt, Germany).

The following antibodies were used in this study: CTSB (CST, #31718), 1:1000 (WB), CTSD (CST, #2284), 1:1000 (WB), GAPDH (Calbiochem, #CB1001, Darmstadt, Germany) 1:20,000 (WB), MAP1LC3B (Thermo Fisher Scientific, Waltham, MA, USA; # PA1-16930), 1:1000 (WB), LAMP1 (DSHB, Iowa City, IA, USA, # H4A3-s), 1:250 (WB), 1:25 (Immunofluorescence (IF)), STAT3 (Cell Signaling Technologies (CST), #9139), 1:1000 (Western Blot (WB)), STAT3 (phospho Tyr705) (CST #9131), 1:1000 (WB) Stat3 (Santa Cruz, c-20; sc-482), 1:200 (WB), Tubulin (clone DM1A) (Sigma-Aldrich), 1:10,000 (WB).

The following Taqman-Probes were used in this study: APOL2 (Hs01935263_s1), APOL3 (Hs00600896_m1), APOL6 (Hs00229051_m1), BIN1 (Hs00184913_m1), BNIP3 (Hs00969291_m1), BNIP3L (Hs00188949_m1), CTSA (Hs00264902_m1), CTSL (Hs00964650_m1), HIF1A (Hs00153153_m1), MMP1 (Hs00899658_m1), MMP2 (Hs01548728_m1), MMP9 (Hs00957555_m1), NPRL3 (Hs00429221_m1), PLAC8 (Hs00930964_g1), SLC2A1 (Hs00892681_m1), SLC2A3 (Hs00359840_m1), SLC66A1 (Hs01120803_m1), SNAI1 (Hs00195591_m1), TBP (Hs00427620_m1).

### 4.3. SDS-PAGE and Western Blot

Preparation of protein lysates, SDS-PAGE and Western Blotting was performed as described [21]. Briefly, membranes were blocked using 5% BSA/TBS-Tween20 (TBS-T) or 5% Milk/TBS-T for 1 h at room temperature. Primary antibodies were diluted as stated above in 5% BSA/TBS-T or 5% Milk/TBS-T at 4 °C overnight. Secondary goat anti-mouse or goat anti-rabbit (dilution 1:10,000, Li-Cor Biosciences, Bad Homburg, Germany) were incubated at room temperature for 1 h and detection was achieved using a LI-COR Odyssey reader (LI-COR Biosciences).

Digitonin-based fractionation was performed as described previously [38,86]. Briefly, cells were pelleted, resuspended in 50 µL PBS (Gibco) and incubated with 50 µg/µL digitonin for 15 min while rotating. The cytosolic fraction was separated from the lysosomal compartment through differential centrifugation.

### 4.4. Cell-Based Assays

MTT (3-(4,5-Dimethylthiazol-2-yl)-2,5-diphenyltetrazolium bromide) assay was basically performed as described previously [87]. Briefly, 2000 to 3000 cells were seeded into 96-well plates and at the indicated time points MTT reagent (5 mg/mL in PBS, Sigma-Aldrich) was added and incubated for 3 h at 37 °C. After removal of the medium and dissolving of formazon crystals using a mixture of 1M HCL:Isopropanol (ration 1:24 *v*/*v*) absorbance was measured using a Tecan Spark plate reader (Tecan, Grödig, Austria).

For Scratch assays 200,000 to 350,000 cells were seeded into 6-well plates. The next day 10 µg/µL Mitomycin C (Sigma-Aldrich) was added for 2 h to inhibit cell division. Hereafter a “scratch” was applied using a pipet tip and after refreshing of the medium (without Mitomycin C) the starting point (0 h) was captured using a Nikon eclipse TE2000-S inverted fluorescence microscope operated by NIS Elements AR version 3.2 at 4× magnification. After the indicated incubation times the same regions were examined again and migrating cells were manually counted with the Cell Counter tool from FIJI [88].

Tumor cell invasion was determined using the Cultrex^®^ 96 Well 3D Spheroid BME Cell Invasion assay (Trevigen, Gaithersburg, MD, USA) according to the manufacturer’s instructions. Therefore, 3000–5000 cells were seeded in U-bottomed 96 well plate in 4 °C cold 1× Spheroid Formation Extracellular Matrix (ECM). Samples were centrifuged at 200× *g* for 3 min at RT to center the cells. Afterwards, samples were incubated for 72 h under culture conditions to allow spheroid formation. Next, the whole plate was chilled for 15 min on ice. Continuously working on ice, 50 µL Invasion Matrix were added to each sample followed by centrifugation at 300× *g* for 4 min at 4 °C. To promote formation of the Invasion Matrix gel, samples were incubated for 1 h at 37 °C and 5% CO_2_. Subsequently, 100 µL FBS-containing medium was added. The spheroids were directly analyzed from 0 h to 48 h with Eclipse TS100 inverted fluorescence microscope using 4× magnification and NIS Elements AR software (version 3.22). Quantitation of the area invaded by the tumor cells occurred with Fiji [88]. Therefore, the threshold to discriminate spheroids from background was adjusted manually and the total area was measured (minimum particle size: 50 pixel^2^). To exclude area changes due to proliferation or spheroid core shrinkage as it may be the case in highly invasive cells [89], the core size was measured and subtracted from the whole area.

### 4.5. Flow Cytometry-Based Assays

All flow cytometry-based application were measured on a BD FACS Aria II or a BD Accuri C6 (BD Biosciences, Franklin Lakes, NJ, USA), per sample 10,000 cells were analyzed.

Annexin V/Propidium Iodide (PI) assay was performed as described previously [82]. Briefly, cells were trypsinized, pelleted and resuspended in FACS-buffer (10 mM HEPES/NaOH pH 7.4, 140 mM NaCl, 5 mM CaCl_2_) containing Annexin V-APC (BD Biosciences) or Annexin V-Fluos (BD Biosciences) and PI and measured within 2 h.

EGFP-LC3-mRFP-LC3ΔG [54] fluorescence was measured on a BD Accuri C6 after the cells were harvested and resuspended PBS. The cells were measured in the FL-1 (EGFP) and FL-3 (mRFP) channels of a BD Accuri C6 and the ratio of EGFP/mRFP was calculated to estimate the autophagic flux.

Lysotracker^TM^ Deep Red (excitation/emission max 647/668 nM; Thermo Fisher) was used according to the manufacturer’s instruction. Briefly, 30 min prior to cell harvesting 25 nM LTDR was added and incubated at 37 °C in the dark. After trypsination, the cells were pelleted and washed twice with PBS and finally resuspended in 50 µL PBS for measurement in the FL-4 channel of a BD Accuri C6.

### 4.6. Immunofluorescence Microscopy

Standard immunofluorescent stainings were performed as described previously [87] using 8-well-chamber slides (Falcon, Corning, Amsterdam, NY, USA) and Alexa Fluor^®^-coupled secondary IgG antibodies (Thermo Fisher).

To monitor the autophagic flux using stable transfectants of ptf-LC3 [53] the cells were treated as indicated and hereafter fixated using 4% paraformaldehyde (PFA, Santa Cruz) for 20 min at RT. After three washing steps with cold PBS the slides were briefly rinsed with de-salted H_2_O and mounted DAPI containing immunoselect antifading mounting medium (Dianova, Hamburg, Germany).

Cellular cholesterol was stained using the cell-based cholesterol assay kit (Abcam Biochemicals) including filipin III based on the manufacturer’s instructions. Afterwards the slides were prepared for standard immunofluorescent stainng against LAMP1 as described above.

To monitor leaky lysosomes stable transfectants of pmCherry-Gal3, excitation/emission max 586/610 nm were treated as indicated and further processed for standard immunofluorescence staining as described above.

All images were acquired using a Nikon eclipse TE2000-S inverted fluorescence microscope operated by NIS Elements AR version 3.2 (both Nikon, Tokyo, Japan).

### 4.7. Whole Transcriptome Analyses

In total, 50–100 ng of total RNA was fragmented by addition of 5× fragmentation buffer (200 mM Tris acetate, pH 8.2, 500 mM potassium acetate and 150 mM magnesium acetate) and heating at 94 °C for 2 min in a thermocycler followed by ethanol precipitation with ammonium acetate and GlycoBlue (Life Technologies) as carrier. Fragmented RNA was further processed using the Ovation Human FFPE RNA-Seq Library Systems (Tecan) according to the instructions of the manufacturer. This library preparation includes reverse transcription using random priming, second strand synthesis, blunt end repair, adapter ligation with nucleotide analog-marked adaptors, strand selection and insert dependent adapter cleavage (inDA-C) to remove rRNA, globin, and other house-keeping transcripts. Target sequences for inDA-C were derived from human sequences. The barcoded libraries were purified and quantified using the Library Quantification Kit—Illumina/Universal (KAPA Biosystems; Sigma-Aldrich). A pool of up to 10 libraries was used for sequencing at a concentration of 10nM. Sequencing of 2 × 150 bp was performed with an Illumina NextSeq 550 sequencer at the sequencing core facility of the IZKF Leipzig (Faculty of Medicine, University Leipzig) according to the instructions of the manufacturer. Demultiplexing of raw reads, adapter trimming and quality filtering was done according to Stokowy et al. [90]. Mapping of the remaining reads against the human reference genome (hg38) was done using TopHat 2 and Cufflinks 2 [91,92].

To analyze biologically relevant signaling pathways we employed the PANTHER-tool (http://www.pantherdb.org/; v14.1, accessed on: 7 May 2019) [56,93] to perform an enrichment analysis. For this purpose all gene names together with their log2 fold-change in gene expression after STAT3-KO were imported to PANTHER and significantly enriched pathways were selected.

### 4.8. Taqman-Based Gene Expression Analyses

RNA-isolation and cDNA-synthesis was performed as described previously [87]. Briefly, RNA-Isolation was performed using the ExtractMe Total RNA Kit (Blirt S.A., Gdansk, Poland) and cDNA-synthesis was achieved using the SuperScript III System (Life Technologies, Darmstadt, Germany) according to the manufacturers instruction using 100 U SuperScript per sample. The quantitative Real-Time PCR (qRT-PCR) was performed using 2× Fast-Start Universal Probe Master Mix (Roche, Risch, Switzerland) and 20× Taqman Probes (Applied Biosystems, Darmstadt, Germany) on a StepOne Plus System (Applied Biosystems) using the standard setting. The target gene expression values were normalized to the reference gene TATA-box binding protein (TBP).

## 5. Conclusions

Here, we present a novel pro-death function of the oncoprotein STAT3, which is frequently overactivated in multiple human cancers including glioblastoma and is associated with the most aggressive mesenchymal subtype. Using isogenic Crispr/Cas9-Knockouts and stable shRNA-mediated STAT3-KD cells we show that loss of STAT3 leads to reduced sensitivity to known ACD-inducers. We show further that this is accompanied by reduced lysosomal lipid accumulation in STAT3-deficient cells and therefore less leakage of lysosomal contents in the cytosol, which causes LDCD in STAT3-proficient cells. In conclusion, these findings offer new research directions to develop targeted therapies of STAT3-activated, autophagy-proficient tumors such as glioblastoma.

## Data Availability

Deep sequencing datasets generated and analysed during the current study are available in the Gene Expression Omnibus (GEO) repository, accession number GSE162429.

## Supplementary Materials

The following are available online at https://www.mdpi.com/article/10.3390/cancers14020339/s1, Figure S1. STAT3 expression and activation across 12 GBM cultures. Figure S2: Functional Validation of Crispr/Cas9-mediated STAT3-Knockouts. Figure S3. Stable shRNA-mediated depletion of STAT3 protects GBM cells from autophagy-dependent cell death. Figure S4. Stable overexpression of STAT3in STAT3-deficient cells restores sensitivity to Pimozide-induced cell death. Figure S5. Mesenchymal glioblastoma stem-like cells are more sensitive to Pimozide-treatment compared to proneural GSCs. Figure S6. Depletion of STAT3 does not change autophagic flux at the level of autophagosome-lysosome-fusion. Figure S7. Transcriptomic analyses after STAT3 depletion in MZ-54-cells reveal changes in lipid metabolism and validation of selected target genes using STAT3-KD cells. Figure S8. Correlation of STAT3 with selected candidate genes from TCGA data. Figure S9. Depletion of STAT3 reduces lysosomal cholesterol-accumulation and lysosomal acidification. Table S1: Correlation of STAT3 expression and selected candidate genes from TCGA data. Supplementary File: Original Blots.
