# Supplementary material for: STAT3 Enhances Sensitivity of Glioblastoma to Drug-Induced Autophagy-Dependent Cell Death
Source: Cancers (Basel). 2022 Jan 11;14(2):339. doi: 10.3390/cancers14020339 (PMC8773829; doi:10.3390/cancers14020339)

Related to Figure 1A

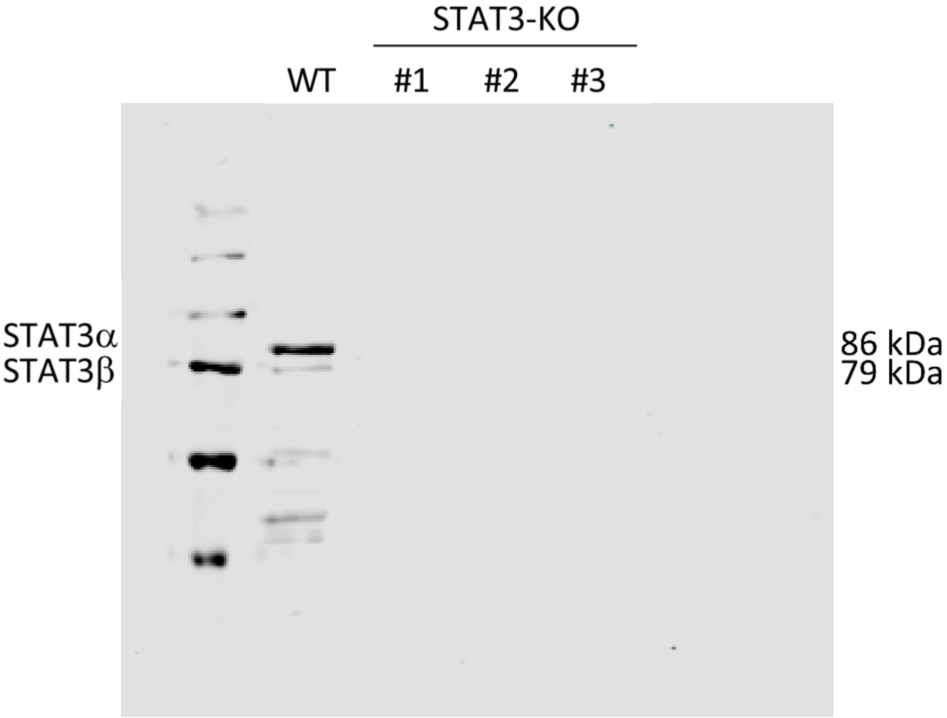

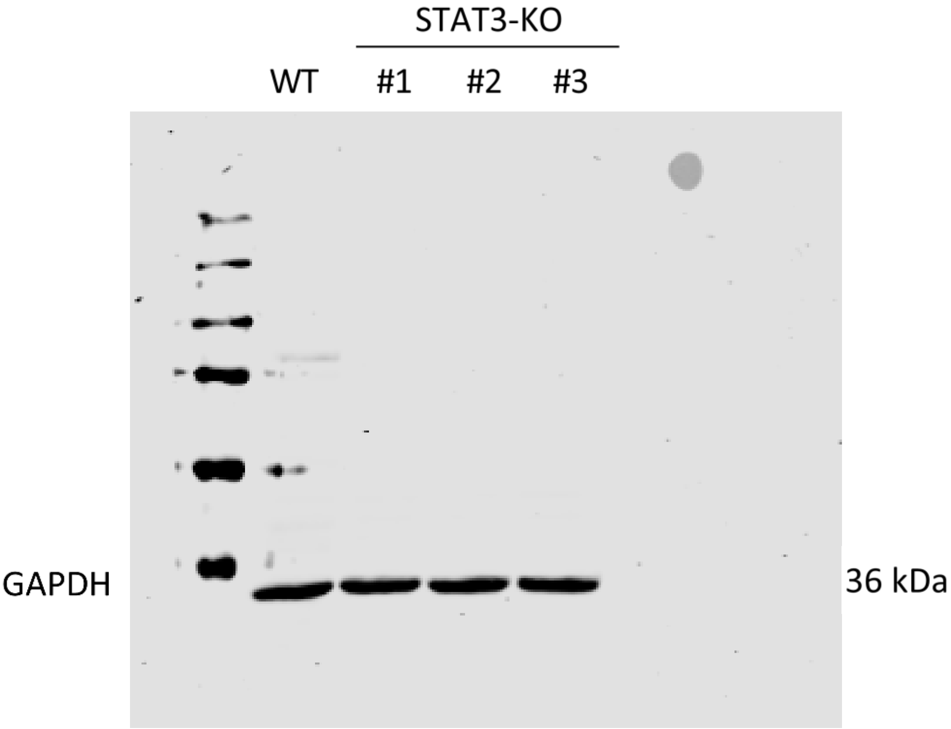

Related to Figure 1F

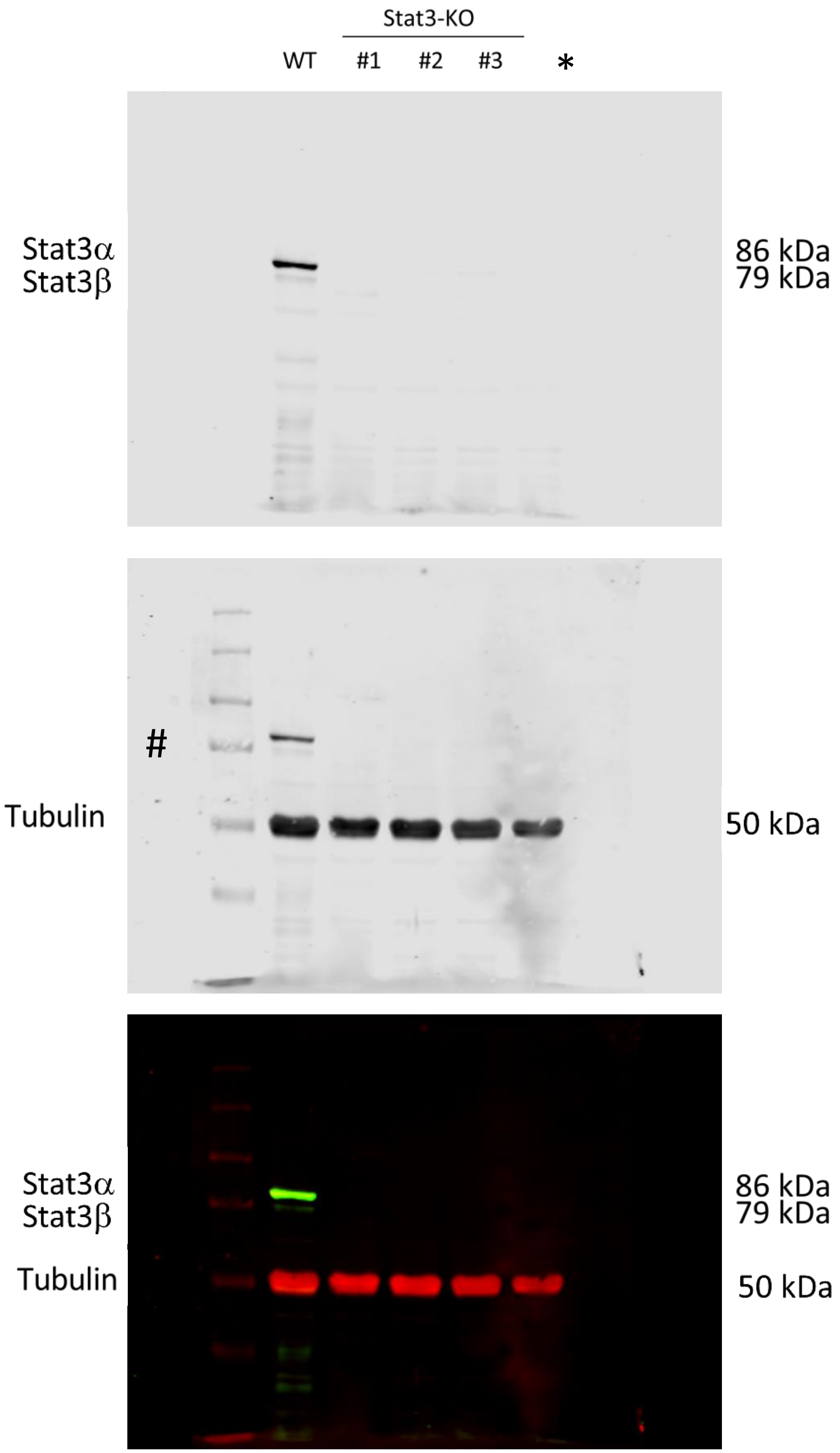

*: Note, that this blot shows an additional Stat3-KO, which was not used in the manuscript

#: Note, that this is the secondary antibody (goat-anti-mouse), detecting tubulin (mouse anti-tubulin) detecting murine Stat3 as well

Related to Figure 1H

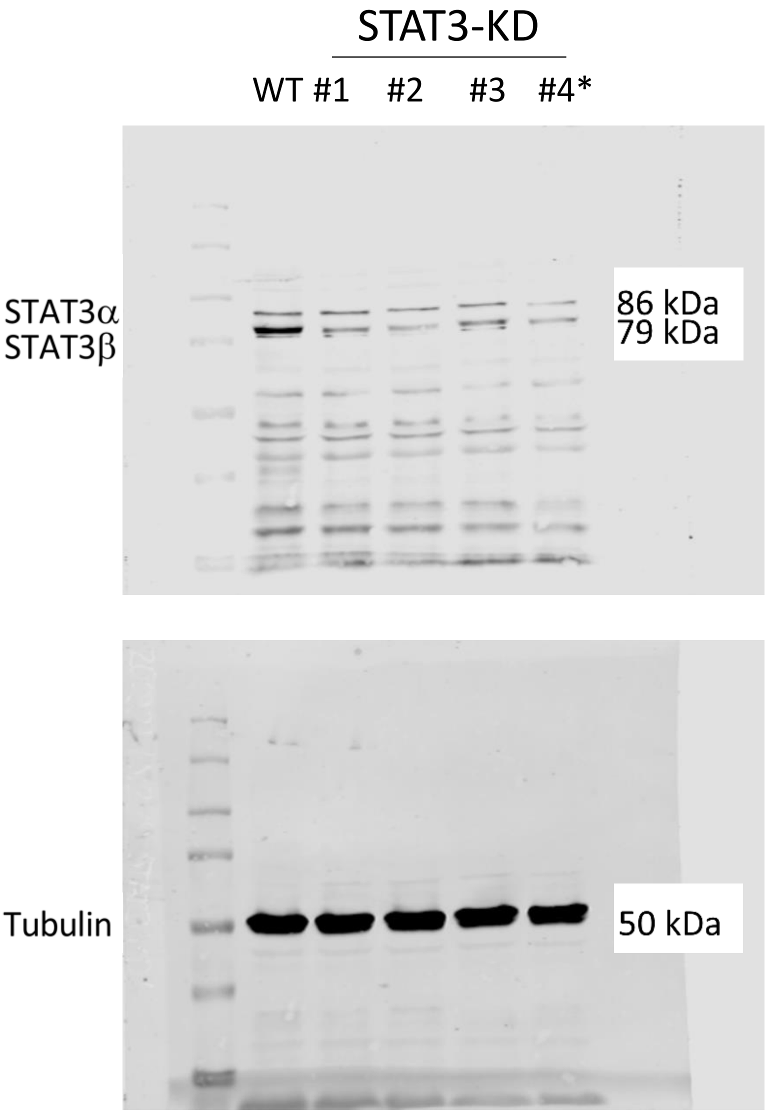

*: Note, that this blot shows an additional Stat3-KO, which was not used in the manuscript

Related to Figure 02

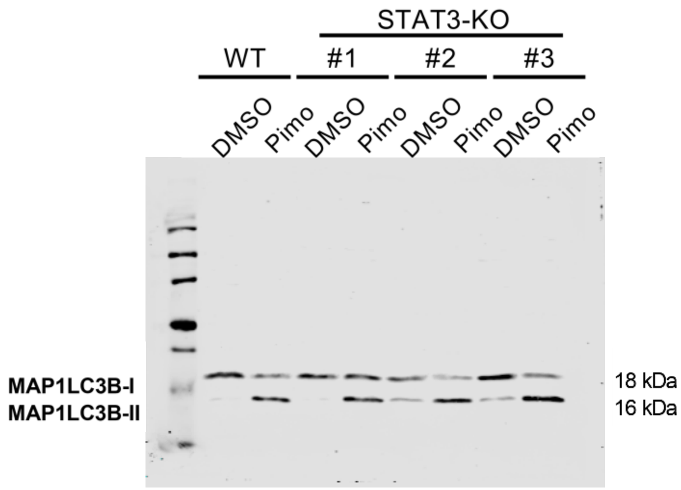

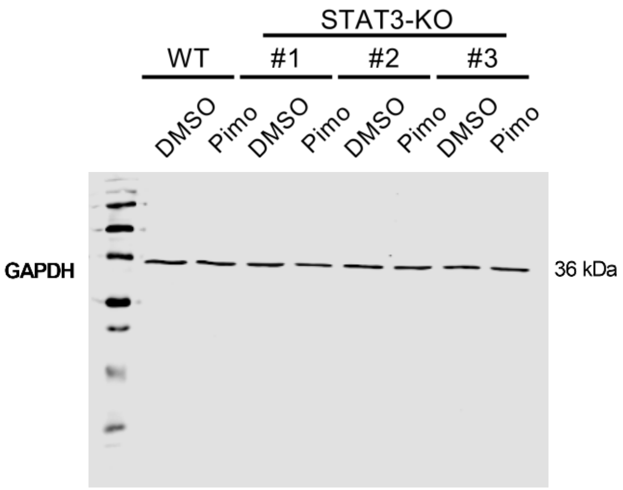

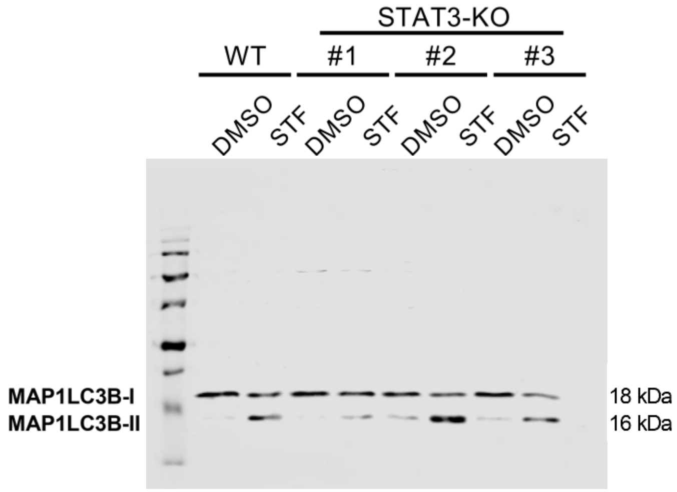

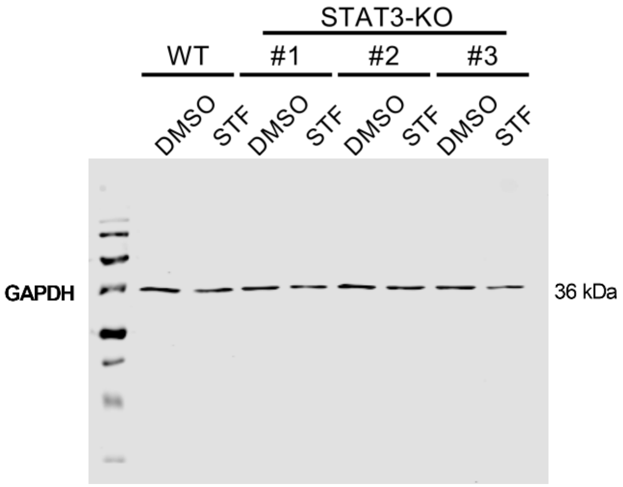

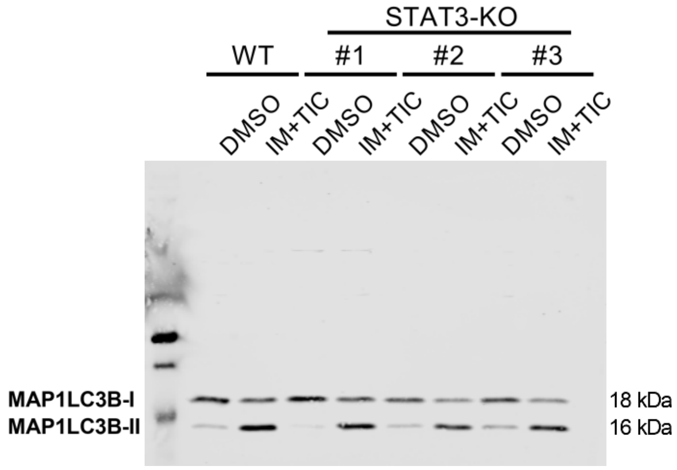

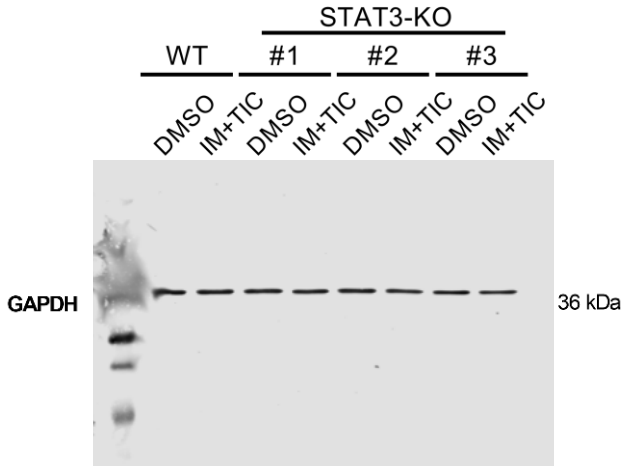

Related to Figure 06

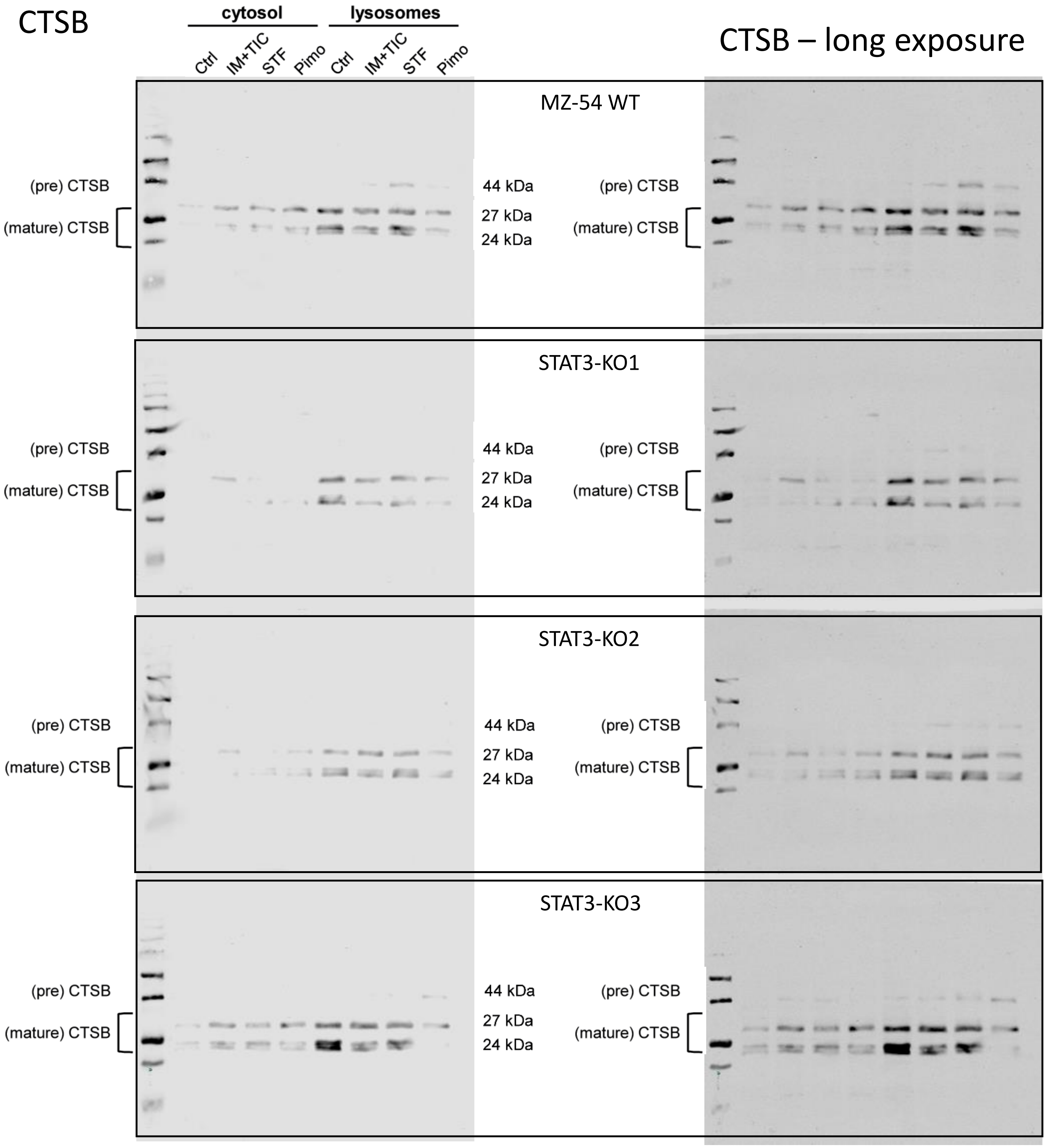

Related to Figure 06

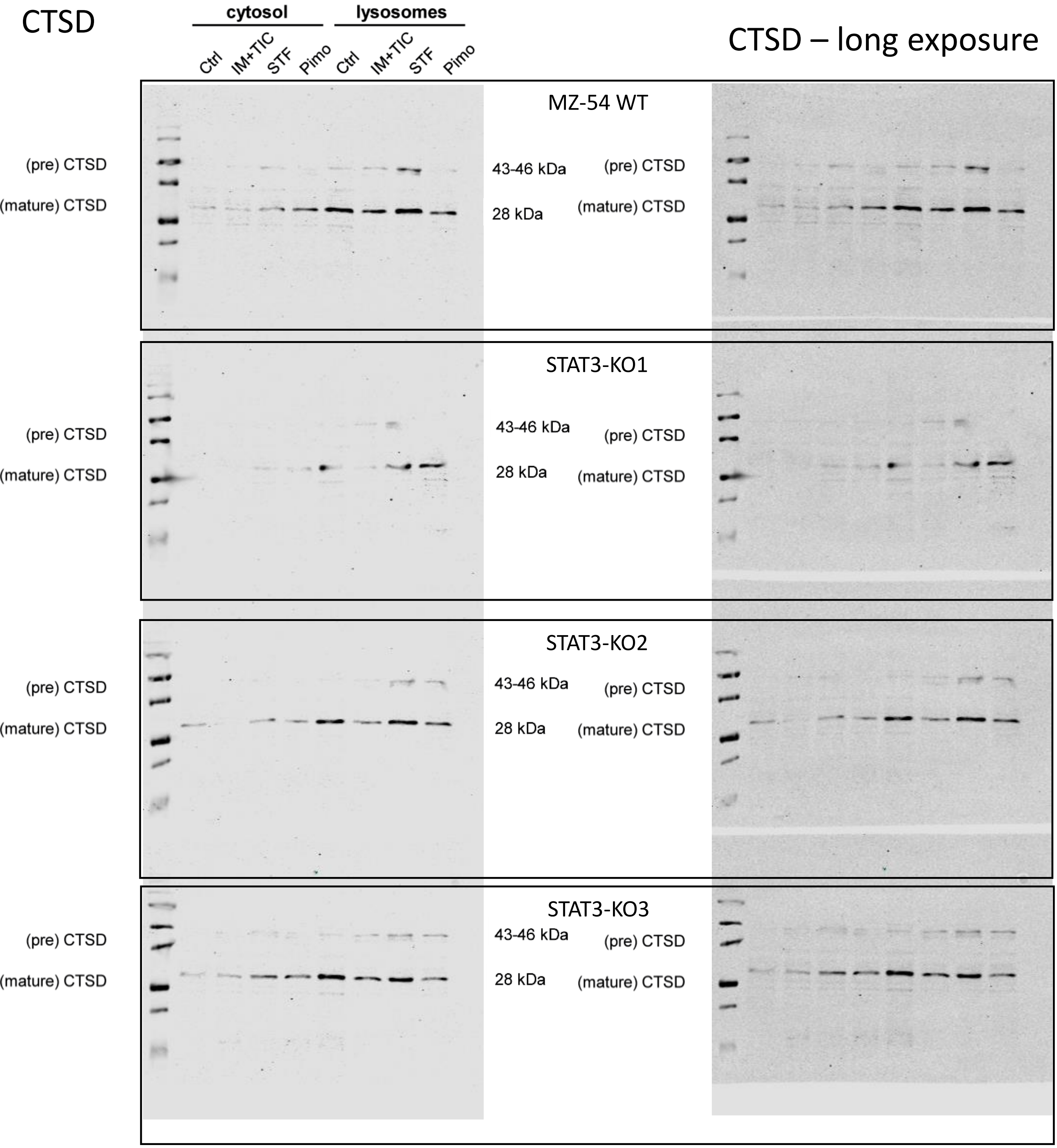

Related to Figure 06

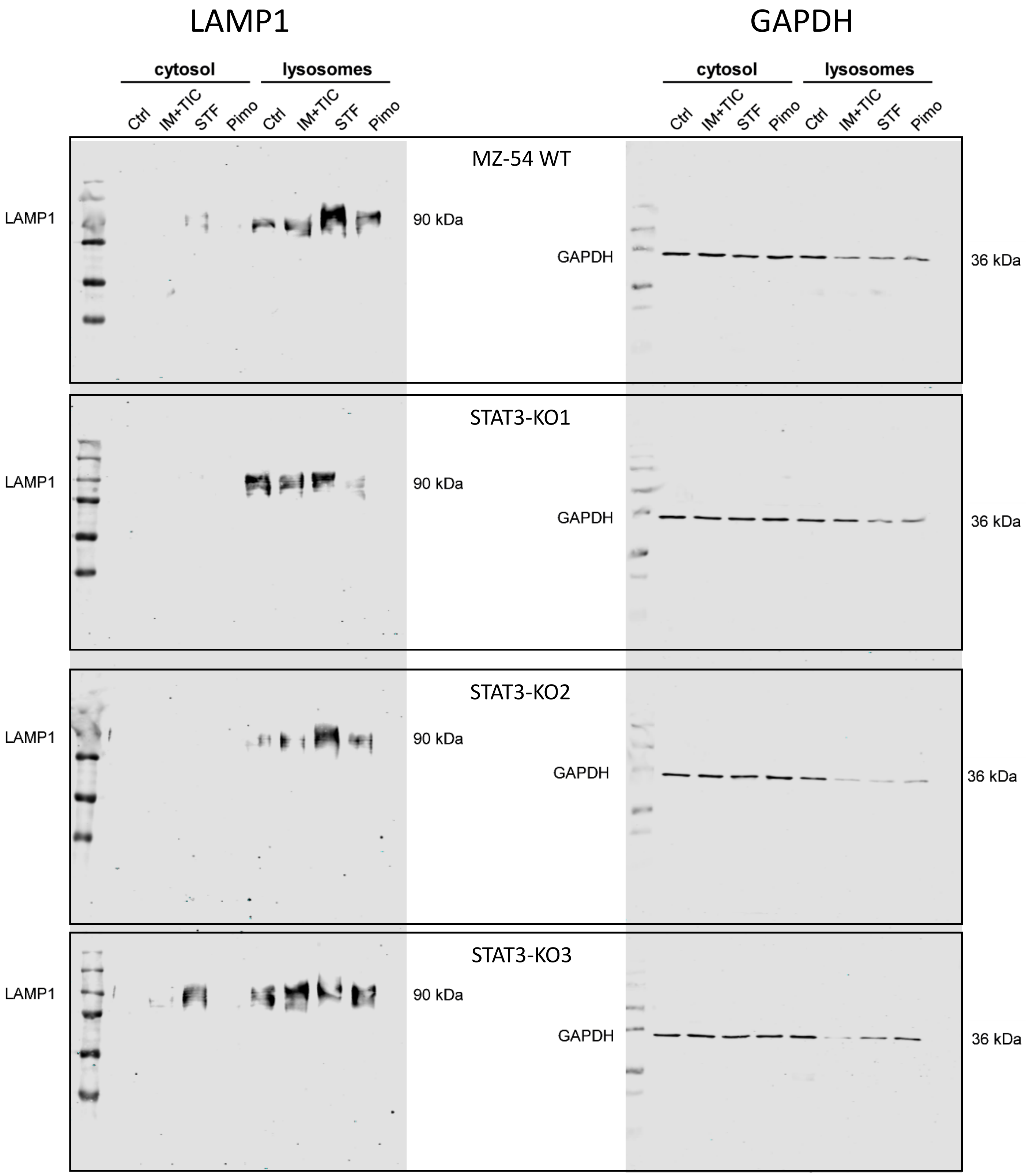

Related to Supplemental Figure 01

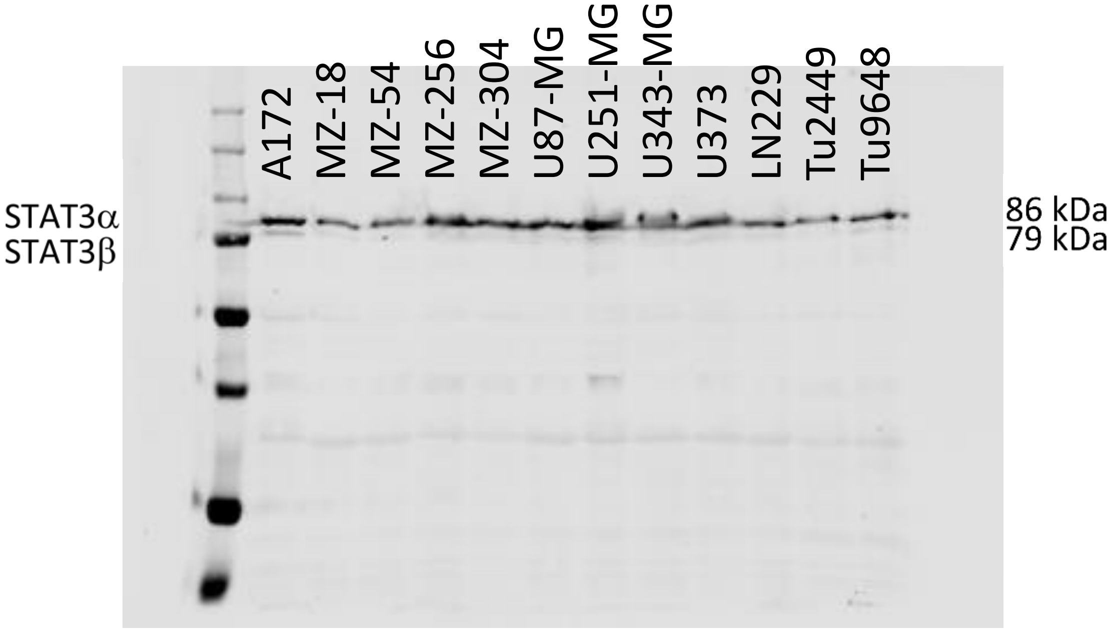

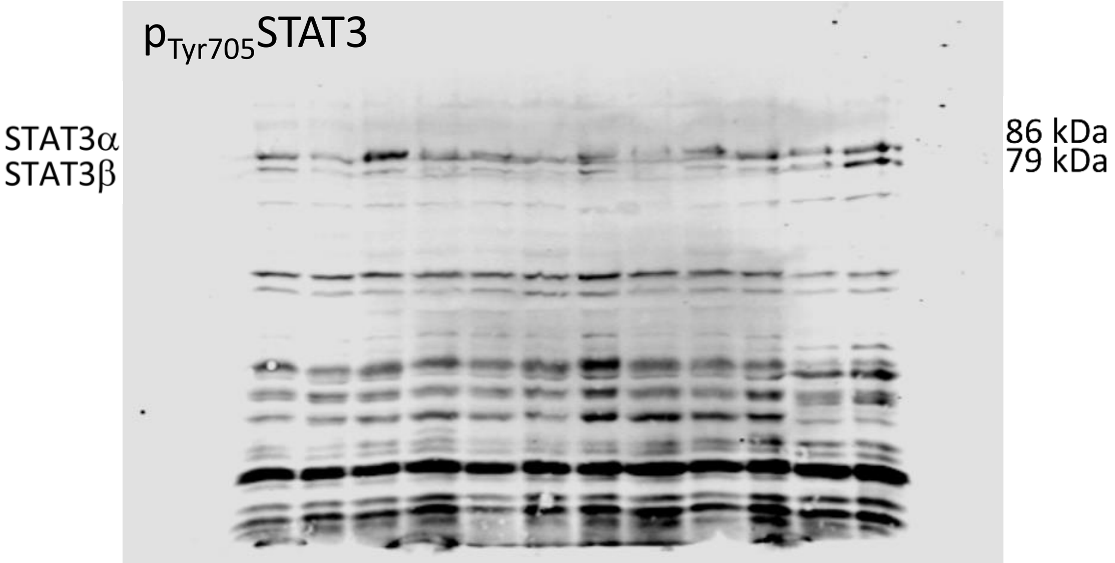

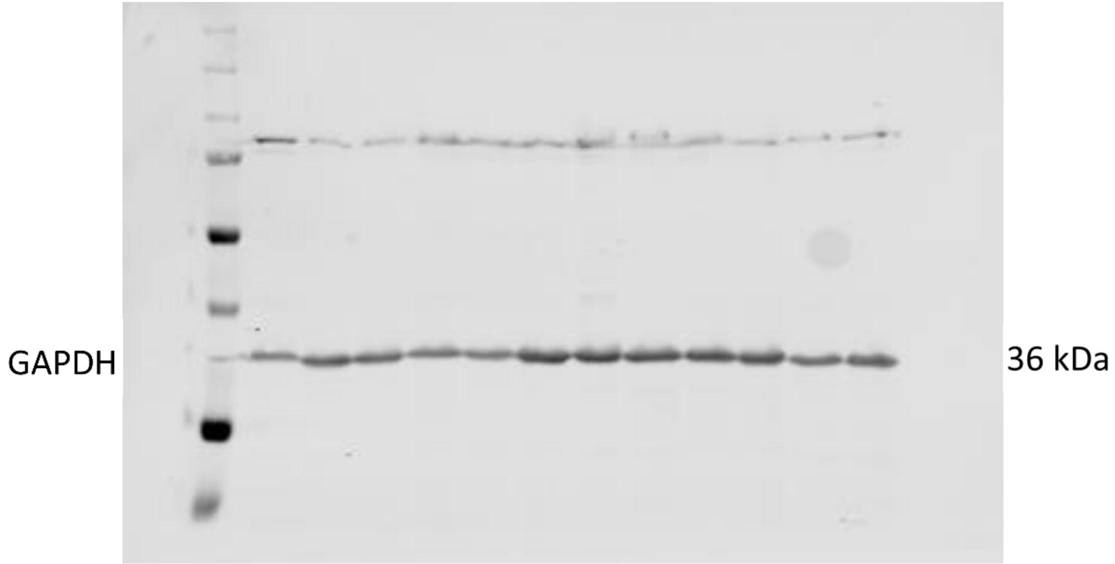

Related to Supplemental Figure 3

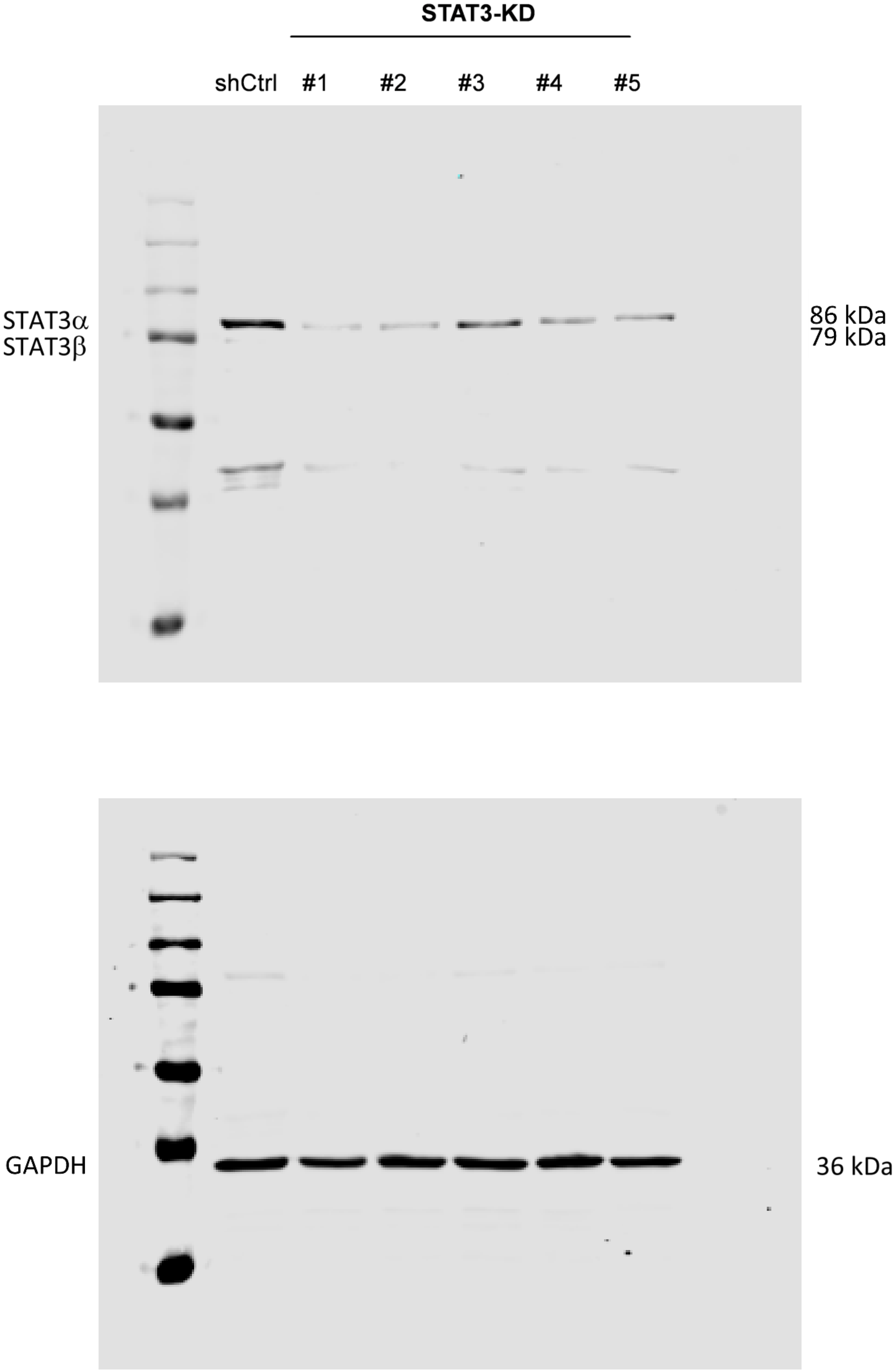

Related to Supplemental Figure 4

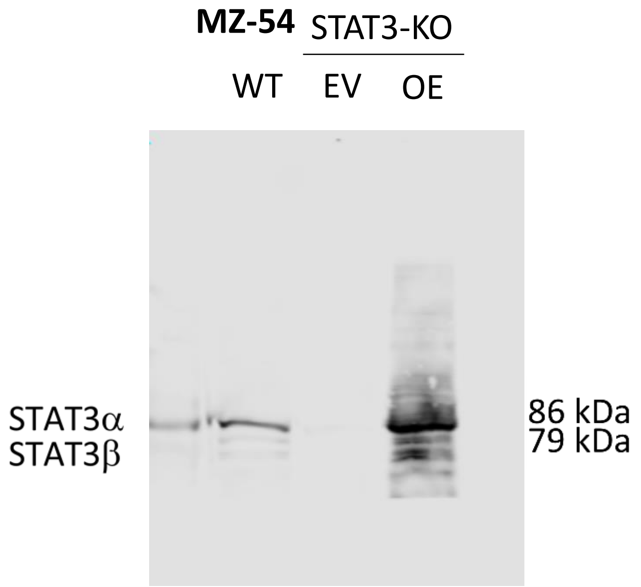

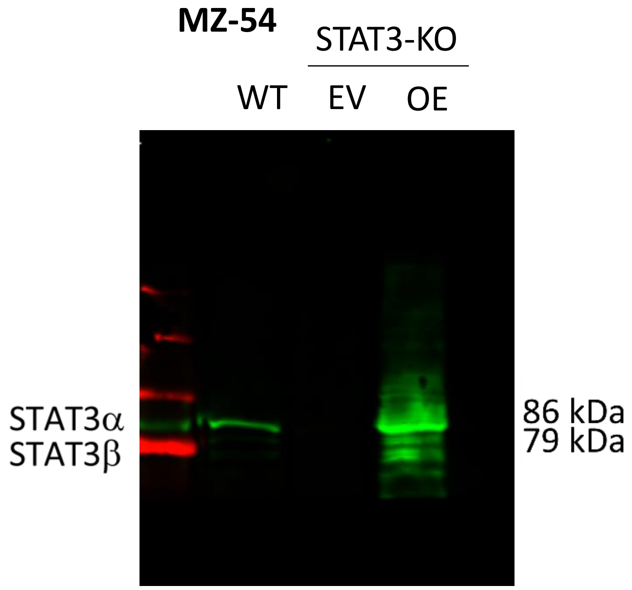

Blot of STAT3 (green) with the marker bands visible (red)

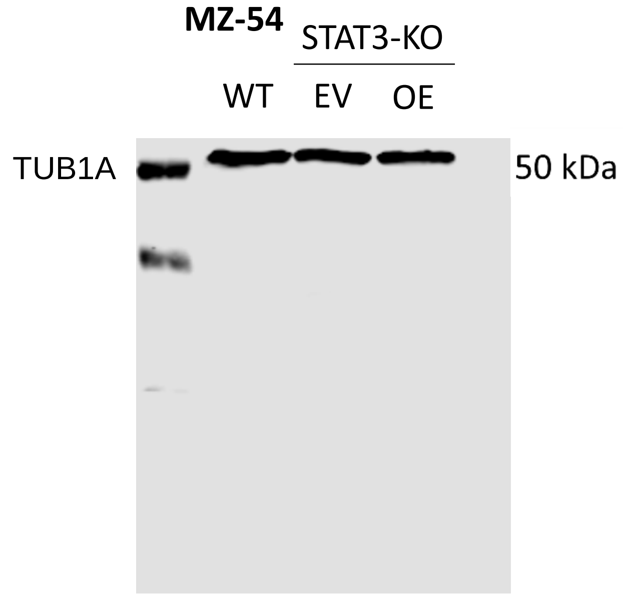

Supplement: Supplementary file 1 [file cancers-14-00339-s001.zip › cancers-1535797-Supplementary File-Original Blots.pdf]
