# Supplementary figures and images for: STAT3 Enhances Sensitivity of Glioblastoma to Drug-Induced Autophagy-Dependent Cell Death
Source: Cancers (Basel). 2022 Jan 11;14(2):339. doi: 10.3390/cancers14020339 (PMC8773829; doi:10.3390/cancers14020339)

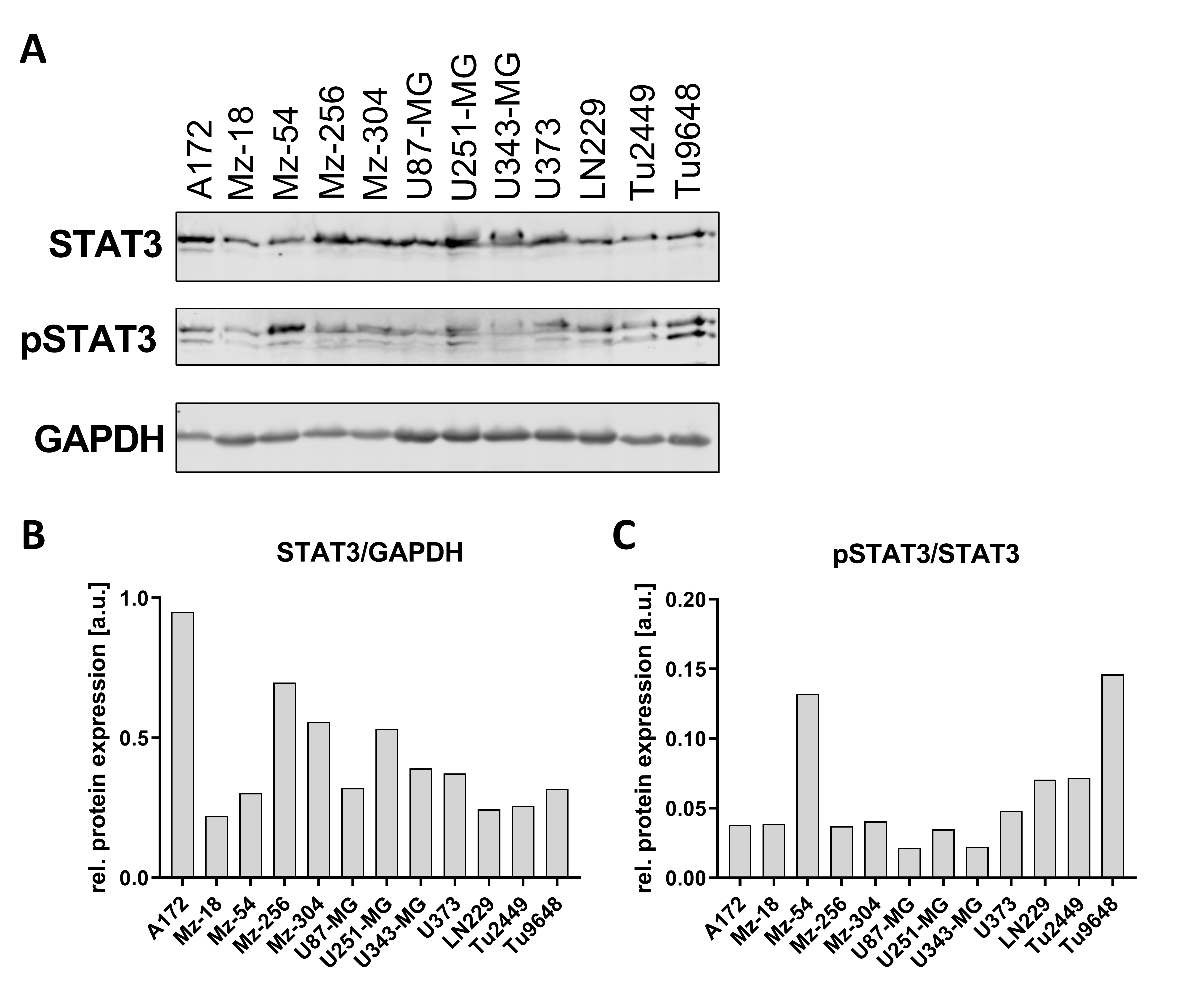

Supplement: Supplementary file 1 [file cancers-14-00339-s001.zip › FigureS1.tif]

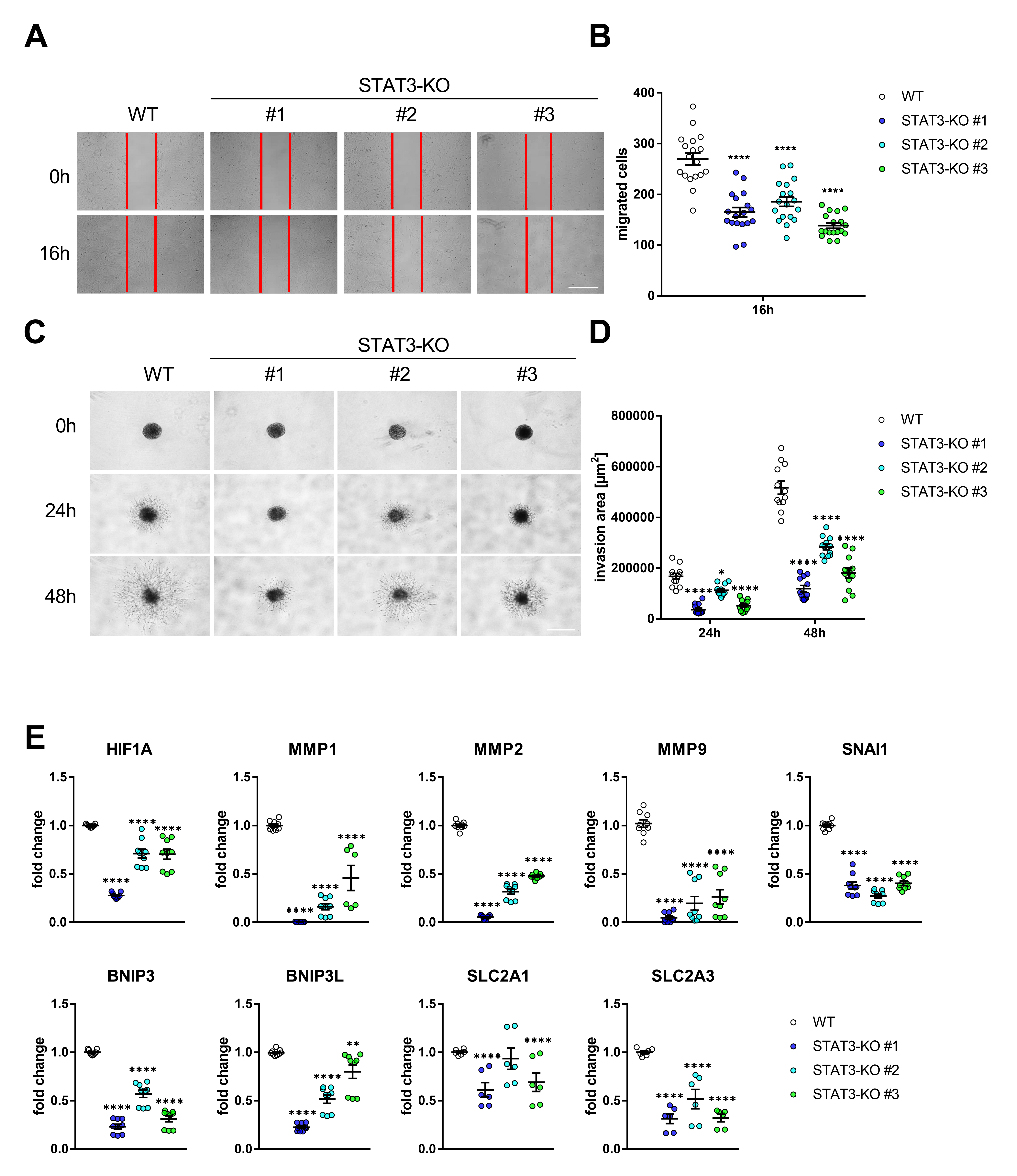

Supplement: Supplementary file 1 [file cancers-14-00339-s001.zip › FigureS2.tif]

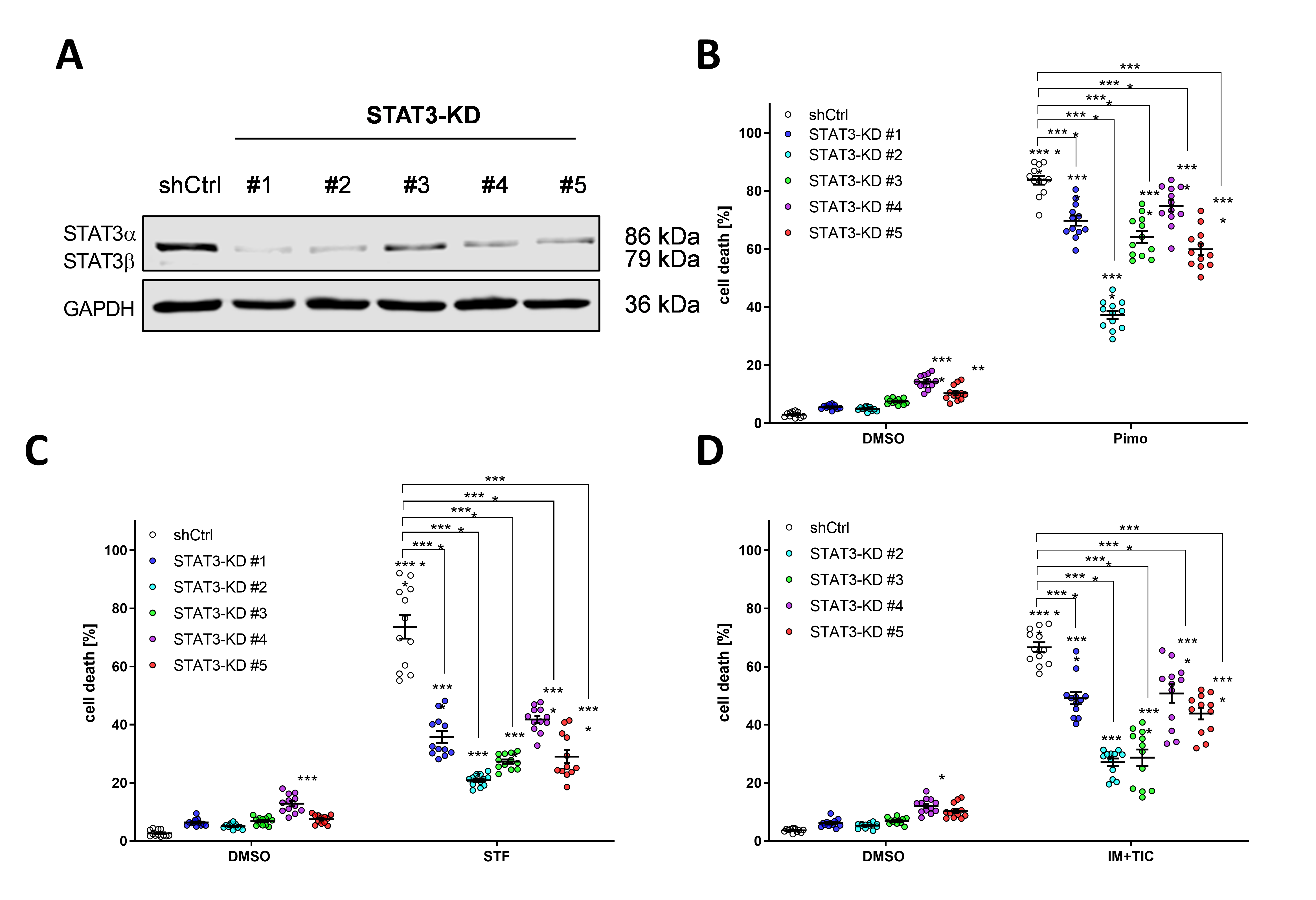

Supplement: Supplementary file 1 [file cancers-14-00339-s001.zip › FigureS3.tif]

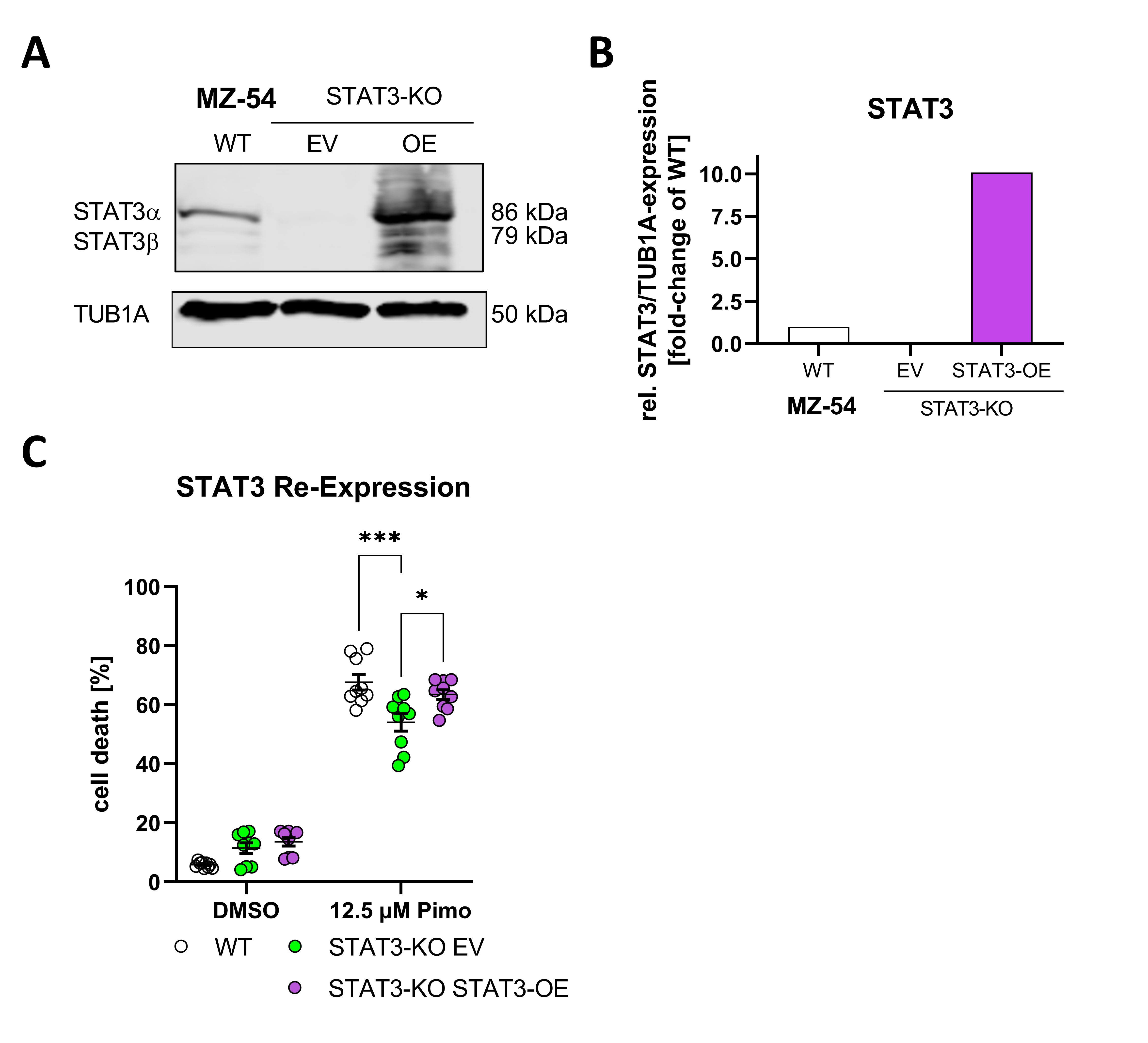

Supplement: Supplementary file 1 [file cancers-14-00339-s001.zip › FigureS4.tif]

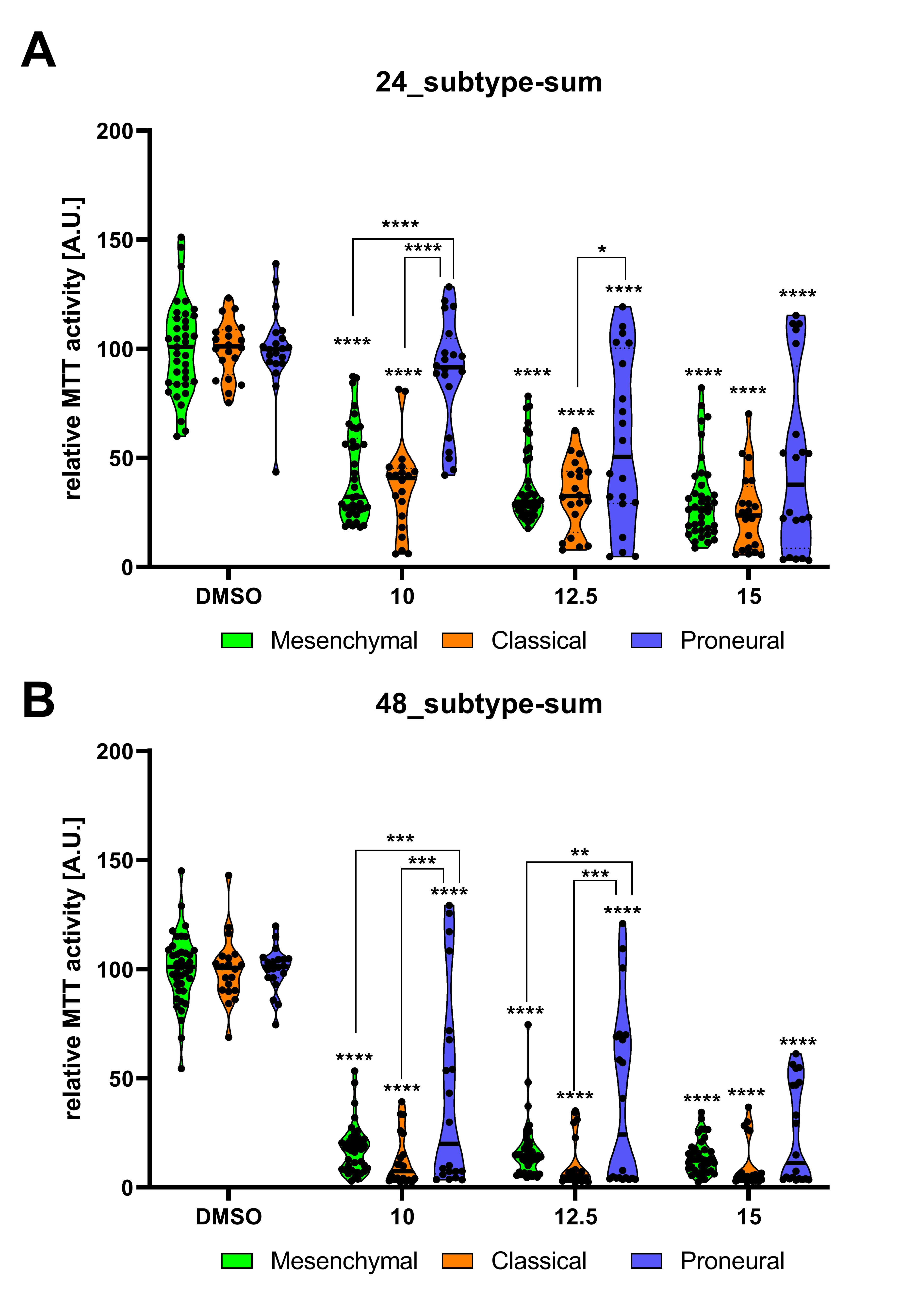

Supplement: Supplementary file 1 [file cancers-14-00339-s001.zip › FigureS5.tif]

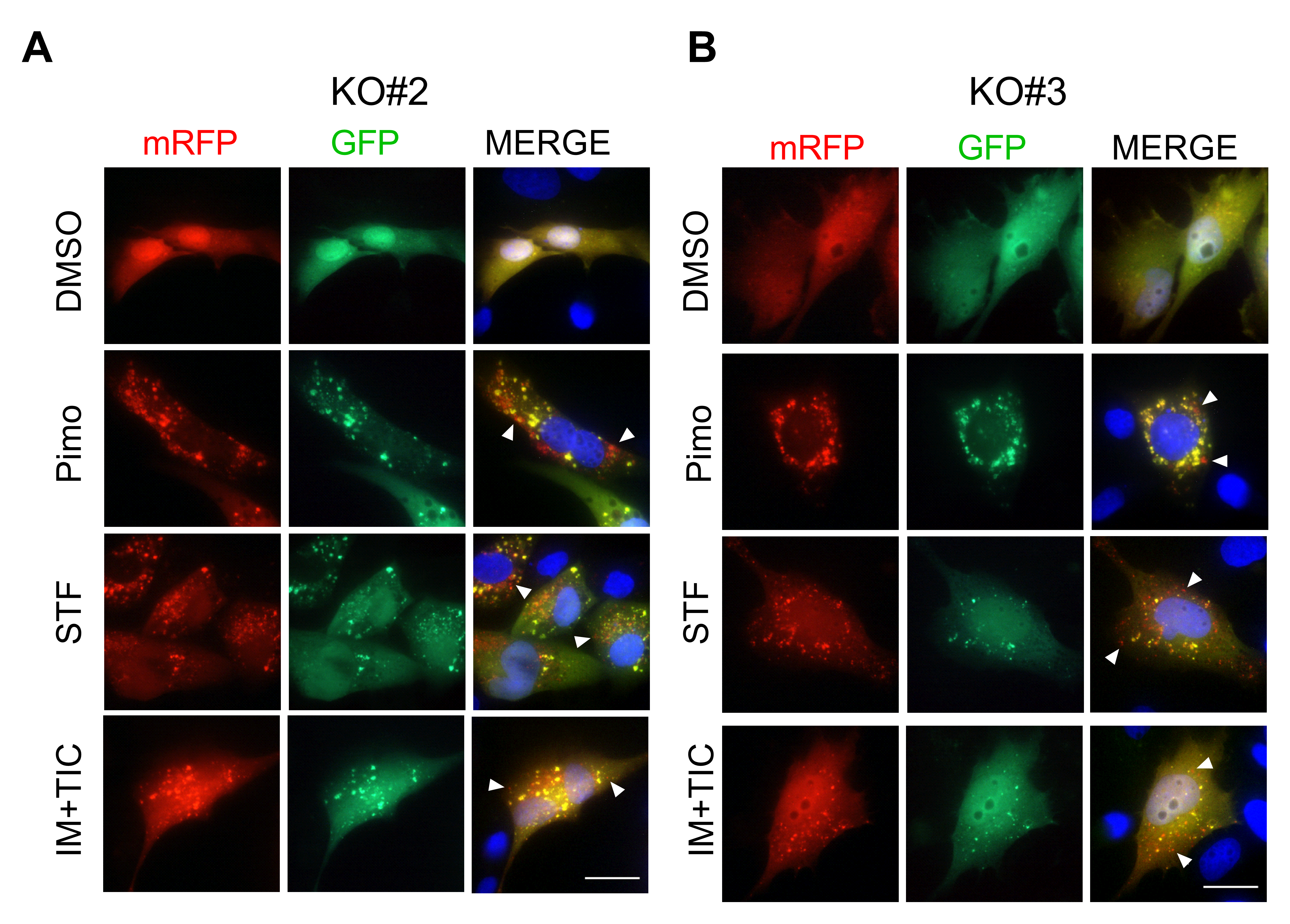

Supplement: Supplementary file 1 [file cancers-14-00339-s001.zip › FigureS6.tif]

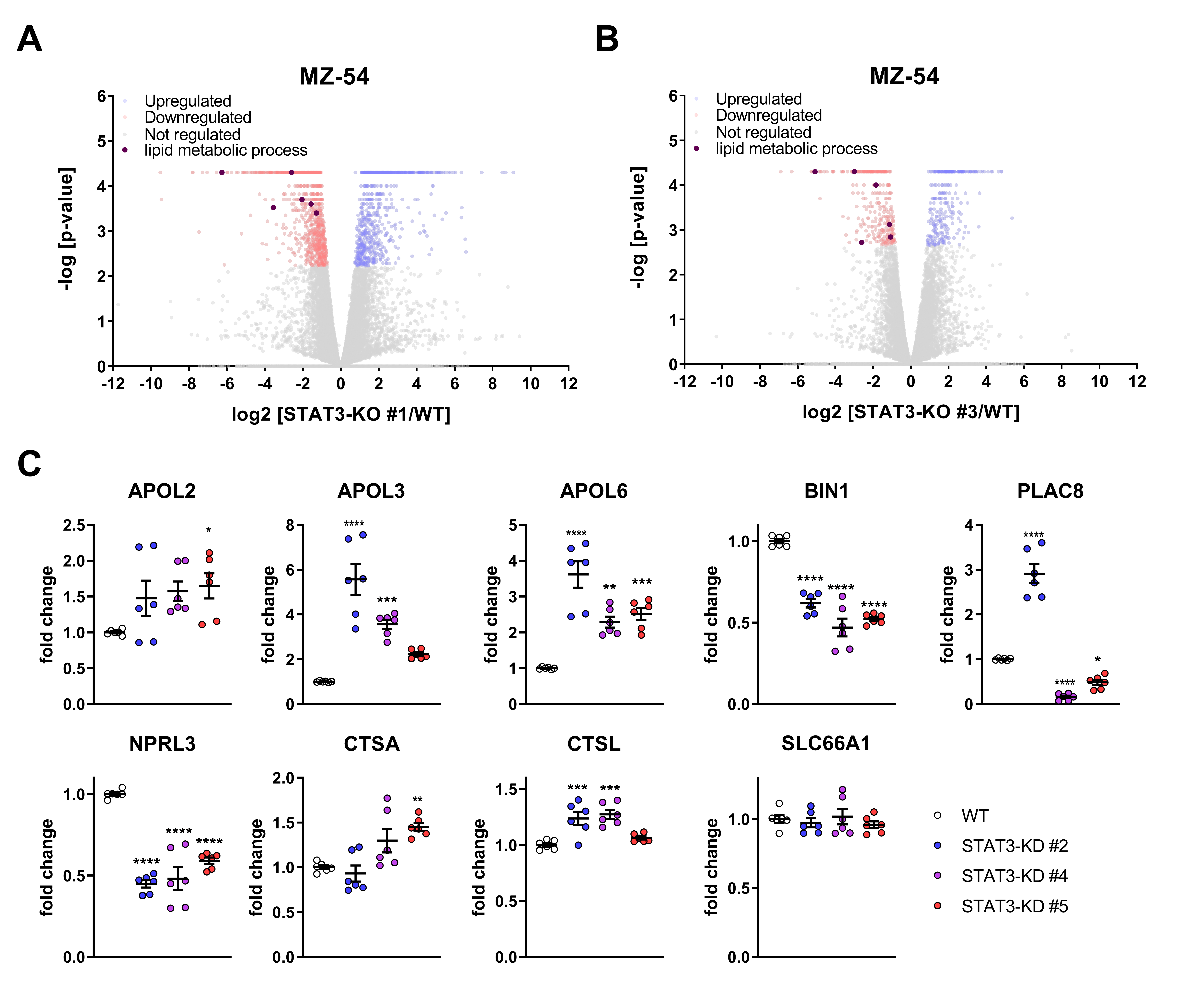

Supplement: Supplementary file 1 [file cancers-14-00339-s001.zip › FigureS7.tif]

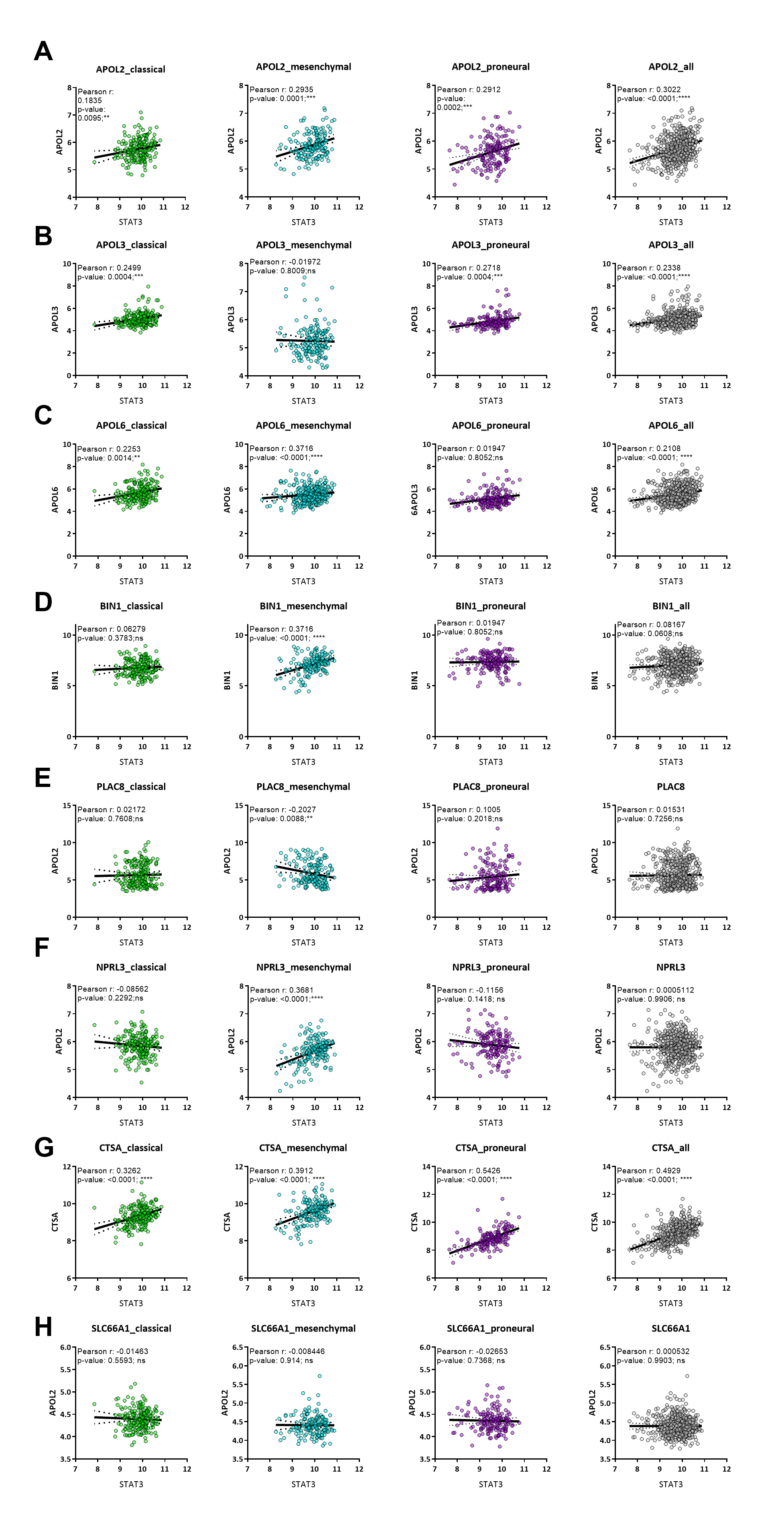

Supplement: Supplementary file 1 [file cancers-14-00339-s001.zip › FigureS8.tif]

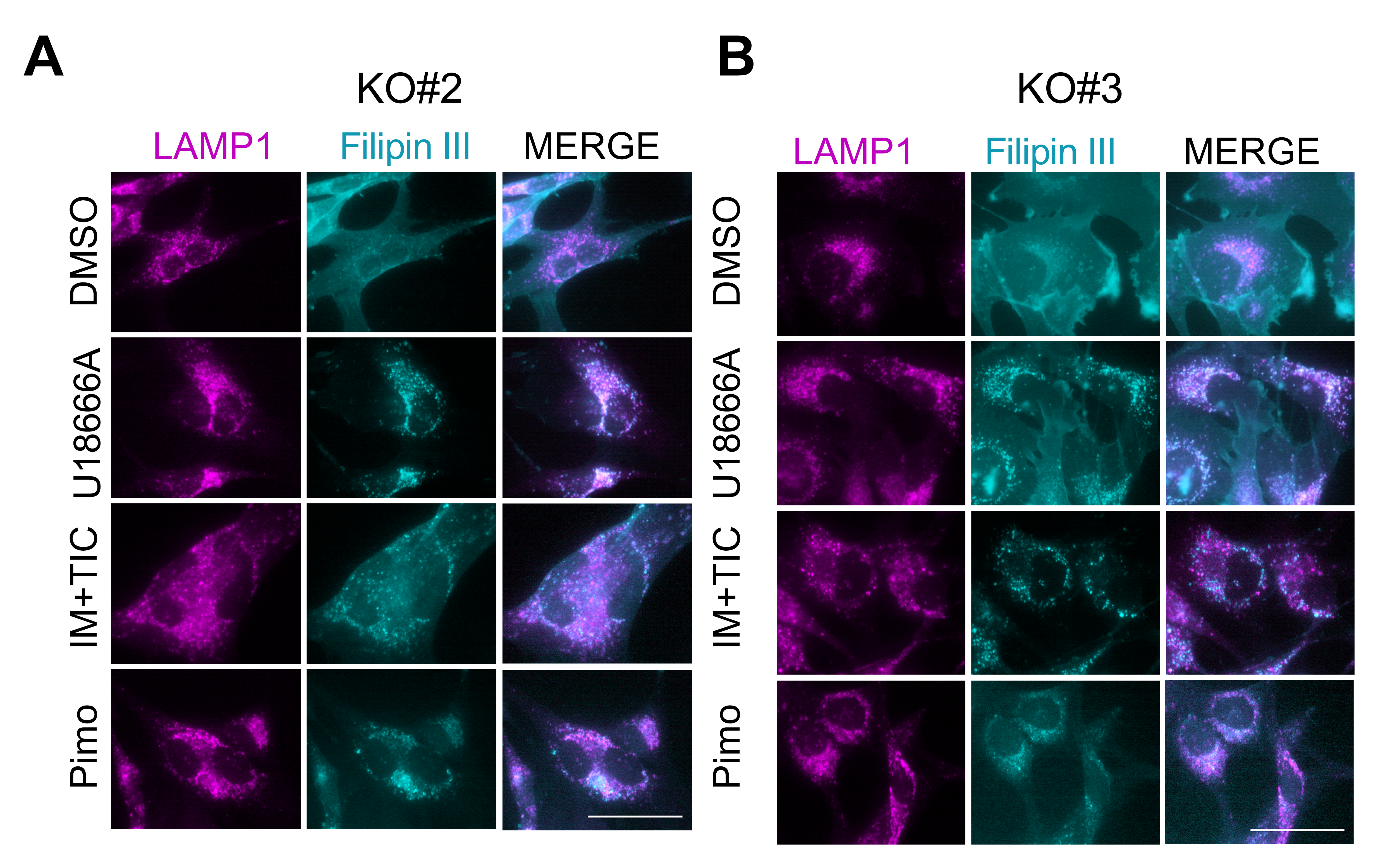

Supplement: Supplementary file 1 [file cancers-14-00339-s001.zip › FigureS9.tif]
